# Relevance of lipoproteins, membranes, and extracellular vesicles in understanding C-reactive protein biochemical structure and biological activities

**DOI:** 10.3389/fcvm.2022.979461

**Published:** 2022-09-08

**Authors:** Lawrence A. Potempa, Wei Qiao Qiu, Ashley Stefanski, Ibraheem M. Rajab

**Affiliations:** ^1^College of Science, Health and Pharmacy, Roosevelt University Schaumburg, Schaumburg, IL, United States; ^2^Department of Pharmacology and Experimental Therapeutics, Boston University School of Medicine, Boston, MA, United States; ^3^Alzheimer’s Disease Center, Boston University School of Medicine, Boston, MA, United States; ^4^Department of Psychiatry, Boston University School of Medicine, Boston, MA, United States

**Keywords:** C-reactive protein (CRP), mCRP, apolipoproteins (apoB), apolipoprotein E, inflammation, pCRP

## Abstract

Early purification protocols for C-reactive protein (CRP) often involved co-isolation of lipoproteins, primarily very low-density lipoproteins (VLDLs). The interaction with lipid particles was initially attributed to CRP’s calcium-dependent binding affinity for its primary ligand—phosphocholine—the predominant hydrophilic head group expressed on phospholipids of most lipoprotein particles. Later, CRP was shown to additionally express binding affinity for apolipoprotein B (apo B), a predominant apolipoprotein of both VLDL and LDL particles. Apo B interaction with CRP was shown to be mediated by a cationic peptide sequence in apo B. Optimal apo B binding required CRP to be surface immobilized or aggregated, treatments now known to structurally change CRP from its serum soluble pentamer isoform (i.e., pCRP) into its poorly soluble, modified, monomeric isoform (i.e., mCRP). Other cationic ligands have been described for CRP which affect complement activation, histone bioactivities, and interactions with membranes. mCRP, but not pCRP, binds cholesterol and activates signaling pathways that activate pro-inflammatory bioactivities long associated with CRP as a biomarker. Hence, a key step to express CRP’s biofunctions is its conversion into its mCRP isoform. Conversion occurs when (1) pCRP binds to a membrane surface expressed ligand (often phosphocholine); (2) biochemical forces associated with binding cause relaxation/partial dissociation of secondary and tertiary structures into a swollen membrane bound intermediate (described as mCRP_*m*_ or pCRP*); (3) further structural relaxation which leads to total, irreversible dissociation of the pentamer into mCRP and expression of a cholesterol/multi-ligand binding sequence that extends into the subunit core; (4) reduction of the CRP subunit intrachain disulfide bond which enhances CRP’s binding accessibility for various ligands and activates acute phase proinflammatory responses. Taken together, the biofunctions of CRP involve both lipid and protein interactions and a conformational rearrangement of higher order structure that affects its role as a mediator of inflammatory responses.

## Discovery and isolation protocols for C-reactive protein from body fluids

This review is focused on the relevance of lipids and lipoproteins to the structures, functions, and bioactivities of C-reactive protein (CRP). From its discovery eighty years ago, CRP has been described as existing in serum in more than one form, with at least one such form having some association with apo B-containing lipoproteins. While more recent studies have primarily focused only on the lipid-free, highly defined serum soluble pentameric protein, the role of lipids in affecting CRP biochemical and immunological characteristics need renewed and updated consideration.

## Purification focused on precipitation methods

A detailed compendium of CRP purification methods and procedures from its discovery in 1941 to current times is presented in the Supplementary material and is summarized in this manuscript as Supplementary Table 1. Of key relevance, apo B containing lipoproteins often co-isolate with CRP, which appears in serum in multiple forms with different electrophoretic mobilities. As described, lipids were often removed from source fluids to facilitate affinity isolation procedures and remove contaminating proteins. The biophysical attributes of highly purified CRP described it as a non-glycosylated, non-covalently associated, homo-pentameric globular protein with each subunit having a calcium-regulated primary binding affinity for phosphocholine (PC) ligand.

## Elaborating on C-reactive protein binding interaction for lipid-associated phosphocholine ligand

Using PC groups immobilized on agarose resin and celite-delipidated ascites fluids spiked with 2 mM calcium and adjusted to pH 8.5, Volanakis et al. (1) found complement proteins C3, C4, C1-INH and C1s co-eluting with CRP, as well as IgG and plasminogen. In studying how isolated CRP could bind membrane-associated phosphocholine, Volanakis and Wirtz (2) proved that only PC groups exposed away from the planar membrane surface were suitable ligands. A natural mechanism to expose such groups occurs when membrane lipids are de-esterified into mono-acyl structures (i.e., lysolecithin) by the action of Phospholipase A2 (PLA2). Lysolecithin adds to membrane curvature and has detergent-like properties, exposing not only PC groups, but apolar regions associated with fatty acyl chain packing. The PLA2 hydrolyzed fatty acid is often arachidonic acid, which enters eicosanoid activation pathways. By exposing PC groups, increasing membrane curvature, and releasing arachidonic acid, PLA2 is a known activator of acute-phase inflammatory processes. The biological responses activated by PLA2 include binding and activating CRP. Quantification of PLA2 in serum has diagnostic value. Compounds that selectively inhibit this enzyme are targets of anti-inflammatory drug development (3, 4).

Besides enzymatic removal of a fatty acyl chain on a phospholipid, PC group exposure and increased membrane surface curvature occur when fatty acyl chains are shortened by oxidative processes as might occur by the action of reactive oxygen species. CRP does bind to oxidatively shortened acyl chain-modified lipid structures (5).

Deacylated or acyl-chain shortened lipids result in increased membrane curvature which plays a role in CRP binding. This concept affects CRP binding not only to plasma membranes, but to its interactions with lipoproteins, which can vary greatly in size and curvature. While each CRP subunit binds a PC ligand as a function of calcium with a weak to modest dissociation value (K_*d*_ = 2–18 μM) (6–9), redundant and simultaneous binding of connected binding sites in the pentameric protein provide additional avidity to elicit biological activation energies sufficient to regulate certain CRP bioactivities. The primary force affecting zwitterionic PC group binding to CRP is the negatively charged phosphate group. This realization led to identifying other structures containing phosphate monoesters (i.e., nucleotides, DNA, RNA, and chromatin) as alternative binding ligands for CRP (10, 11). The positively charged choline group of PC does contribute ∼10-fold increased binding affinity for CRP. A closer examination of cationic molecules as ligands for CRP is included below.

## How apolar lipid regions interact with and affect C-reactive protein structure

When pCRP binds to the surface of an activated plasma membrane, different biochemical forces loosen both electrostatic and hydrophobic interactions holding the pentamer together. Initially, the pentamer swells into an intermediate conformation referred to as mCRP_*m*_ (membrane-associated mCRP), or pCRP*. When sufficient external bonding energy is added, pCRP subunits “flip” to assume a new protein structural energy equilibrium which includes enhanced interactions with apolar lipids zones and lipid raft-directed cholesterol binding (4, 12–14). The membrane-bound form of mCRP has strong pro-inflammatory activation bioactivities which include leukocyte activation and cellular damage (15, 16).

The cholesterol-binding peptide of the CRP subunit is localized to residues 35–47 of the 206 amino acid subunit primary sequence (i.e., the CBP). Exposure of this cholesterol binding cleft extends from the periphery of the subunit surface deep into the subunit interior involving the sole disulfide-linked bond found in the interior of each globular CRP subunit (linking residues C_36_ and C_97_). Of relevance, optimal cholesterol binding to the exposed cleft occurs after reduction of this intrachain disulfide bond, suggesting an added level of regulation is needed to release the full potential of CRP’s pro-inflammatory activities (17). A synthetic peptide of CBP (i.e., V_35_CLHFYTELSSTR_47_) not only inhibited mCRP binding to cholesterol, but its binding to C1q, fibronectin, collagen IV, fibrinogen, apolipoprotein B, and lipoprotein particles (18). The CBP is not associated with the PC binding pocket of each CRP subunit but includes residues (_40_YTE_42_) which contribute to the non-covalent inter-subunit stabilization of the pentamer (19). Exposure of CBP to allow CRP to bind cholesterol and its other defined ligands would require subunit dissociation. In addition to residues _40_YTE_42_, Li et al. (18) identified the _36_CLH_38_ tripeptide as the most important in regulating diverse CRP binding activities. The fact that this reactive sequence includes cysteine, which forms an intra-chain disulfide bond with C_97_ located deep within the globular subunit in the compacted structure, also underscores the importance of subunit dissociation and conformational change to express the bioactivities inherent in the CRP molecule. The fact that reducing the disulfide bond, which relaxes protein tertiary constraints manifest by covalent bonding, will maximally elicit CRP activities, is consistent with significant conformational change as a driving factor for expressing CRP biofunction(s).

Mackiewicz et al. (20), using transmission electron microscopy, showed CRP causes a structural clustering of lipids in nanoparticles and could aggregate LDLs. The initial CRP interaction with lipid particles was calcium-dependent and reversible, but over time, the interaction became permanent and correlated with lipid reorganization. These observations are consistent with a lipid-mediated conversion of pCRP into mCRP.

**FIGURE 1:**
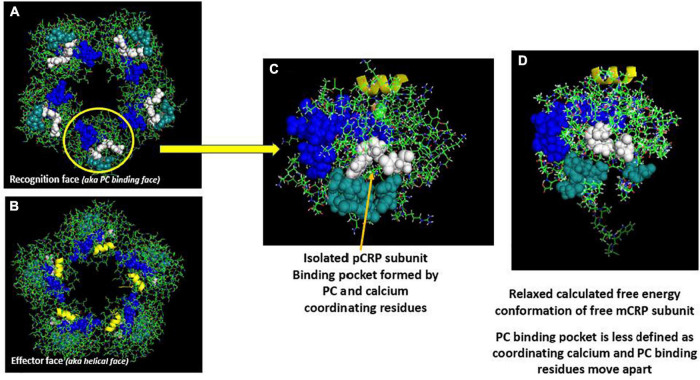
Orientation of calcium-dependent—phosphocholine binding sites and the multi-reactive cholesterol binding peptide (CBP) on serum soluble pentameric CRP (pCRP) and a calculated low energy predictive free CRP subunit (mCRP). **(A)** pCRP (PDB: B109) Recognition face (aka: PC-binding face). **(B)** pCRP flipped 180° to show the Effector face (aka: helical face; helix shown in yellow). Key residues involved in PC binding to CRP subunits: F66, S74, E81, Q150 (Shown in white); Key residues involved in Calcium binding to CRP subunits (2/subunit): D60, N61, E138, Q139, D140, E147, Q150 (shown in dark teal); Defined Cholesterol binding peptide sequence (CBP) on CRP subunits (Residues 35–47: V35CLHFYTELSSTR47 (shown in blue). Note all Calcium and PC binding sites orient on the same face of pCRP such that, when pCRP binds PC, it sits flat on the PC-presenting surface. Key residues in CRP identified in Ji et al. and Pathak and Agrawal (14, 106). **(C)** Isolated pCRP subunit looking down on the PC binding recognition face. Depicted residues are the same as defined in panels **(A,B)**. **(D)** Predicted low free energy structure of the free CRP subunit based solely on its primary protein sequence as described in Xu (42). Note both PC and calcium binding residues rotate away such that the defined binding pocket is altered. Also note the extended peptide sequence shown below the core globular structure. This peptide corresponds to C-terminal residues of CRP (i.e., residues 198–206) that contribute to inter-subunit stabilization in pentameric CRP. A major shift in the orientation of these residues occurs when pCRP subunits dissociate. Of relevance, these residues map to a unique mCRP epitope not expressed in pCRP (21, 107).

**FIGURE 2:**
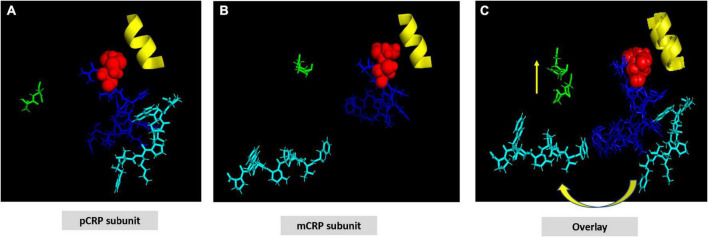
Structural shift of selected residues comparing pCRP subunit and mCRP subunit. **(A)** Shows a pCRP subunit with disulfide bond (C36-C97) shown as red spheres; CRP effector face helix (residues P168-L176) shown in yellow; the cholesterol/multi-ligand binding sequence (CBP: V35-R47) shown in blue; calcium binding residues (D60-N61) shown in green; and the C-terminal octapeptide (F198-P206), which contains residues involved in the inter-subunit contact zone of pCRP pentameric structure shown in cyan. All other residues on the pCRP subunit are hidden to facilitate visualization of the orientation of these described residues. **(B)** Shows these same residues oriented in an mCRP subunit model calculated using low free energy structural algorithms of the CRP primary sequence. **(C)** Overlay of pCRP and mCRP oriented to best overlap the disulfide bond and effector face helix. Note the reorientation of highlighted calcium binding residues and the significant rotation of the inter-subunit contact residues away from the CPB peptide. Shifting the calcium binding residues would affect its stabilizing role in maintaining pCRP’s quaternary structure and in regulating CRP’s binding to phosphocholine ligand. These changes lead to the large rotation of the inter-subunit contact residues allowing ligand access to CPB. Note that the C-terminal octapeptide maps to a unique epitope expressed on mCRP and not pCRP. The structural changes shown here are consistent with increased aqueous exposure of these residues allowing for specific antibody binding.

The nature of CRP’s interaction with lipid was examined using surface tension techniques and various lipid monolayer constructs. While PC-containing lipids could bind pCRP, only mCRP directly inserted into physiologically relevant monolayers that included cholesterol-rich lipid rafts (12, 14). Insertion was dependent on cholesterol content and was mediated by two peptide sequences in CRP, one defined by the CBP described above (i.e., residues 35–47), and one defined by the C-terminal octapeptide (i.e., residues 199–206). This latter sequence localizes to the inter-subunit contact areas that stabilize pentameric CRP and only becomes exposed when pCRP dissociates into mCRP. The octapeptide maps to the unique antigenic epitope expressed on mCRP and the partially dissociated mCRP_*m*_, but not on pCRP (21) (Figures 1, 2).

## Lipoproteins and C-reactive protein

The relevance of lipids to CRP synthesis pathways was described by Kushner and Feldman (22) when liver was identified as the main site of synthesis. Prior to release from hepatocytes into circulation, synthesized CRP was found sequestered in intracellular vesicles that also contained apo B-containing Very Low-Density Lipoproteins (VLDLs), underscoring CRP’s association with lipids begins with post-translational processing and secretion during the early moments of a stimulated acute phase response.

The direct interaction of CRP with lipoprotein particles was described by Saxena et al. (23) using an affinity adsorbent with immobilized rat CRP. Lipoprotein fractions prepared by ultracentrifugation from outdated human blood were passed across this resin and VLDLs carrying apolipoprotein B and apolipoprotein E, and LDLs carrying apolipoprotein B bound the immobilized protein. When unfractionated plasma was used as the source of lipoproteins, similar binding of apo E and apo B lipoproteins were observed, albeit binding of LDLs was much reduced. Binding required calcium and lipoproteins could be eluted using phosphocholine hapten suggesting lipoprotein binding to (rat) CRP involves exposed PC groups. Unlike human CRP, rat CRP is a glycoprotein. Removing sialic acid residues did not affect lipoprotein binding (24). Rat CRP was later shown to also bind apo A1 (HDL) lipoproteins containing cholesterol (25).

In direct LDL binding studies using human CRP, mCRP but not pCRP bound LDL in a calcium independent manner. Binding occurred not only with normal LDLs, but with LDLs oxidized with copper sulfate, and to LDLs both directly adsorbed to, or captured onto a solid phase surface. When purified apo B was used as the binding ligand, both mCRP and pCRP bound, with mCRP exhibiting stronger binding. Binding was inhibited by fluid phase apo B, and by a pentadeca peptide fragment (15-mer) derived from apo B and containing the cationic nonapeptide sequence mediating apo B binding to the LDL-receptor (i.e., apo B sequence #3358-3372 T_3358_**R**LT**RKR**GL**K**LATAL_3372_) (26). The calculated inhibition constant of the pentadecameric peptide was more than 800-fold stronger than that of intact apo B protein and was not influenced by calcium (12). These data show both pCRP and mCRP bind cationic ligands in a way distinct from how calcium regulates CRP binding to phosphocholine ligands.

As the cationic sequence in apo B effecting both CRP and LDL-R binding contains both arginine residues (3) and lysine residues (2), site-directed group modification studies were performed to examine which cationic groups were primarily responsible for interactions with CRP. Modifying apo B lysine residues using aceto-acetylation had no effect, but modifying apo B arginine residues with 1,2, cyclohexanedione reduced binding to rat CRP by ∼70% (25). These data, along with studies of human CRP and rabbit CRP (27–31), indicate CRP has a selectively stronger binding affinity for arginine-containing ligands than lysine-containing ligands (32, 33).

## Other binding interactions of C-reactive protein with cationic ligands

The complement protein C1q, cationic protein with a pI of 9.3, often co-isolates with CRP. It directly binds CRP on the effector face of the pentameric disc (i.e., the opposite face from the PC-binding recognition face) (16) and mediates activation of the classical complement pathway. CRP also binds polycations such as poly-arginine, poly-lysine, and protamine, and positively charged liposomes (33–37) in a way that affects CRP’s effects on both classical and alternative complement pathways (35, 36, 38). While initial CRP-complement studies interpreted CRP’s activities as a protein of rigid structure forming immune-complex-like aggregates that regulate its bioactivities, the more recent awareness and understanding of its distinctive structural isoforms has led to the understanding that the capacity of CRP to bind cationic ligands and complement proteins is enhanced when CRP is in its modified conformation (mCRP) (12, 15, 39, 40).

## Salt bridges and peptide sequences involved in C-reactive protein structural packing

**FIGURE 3:**
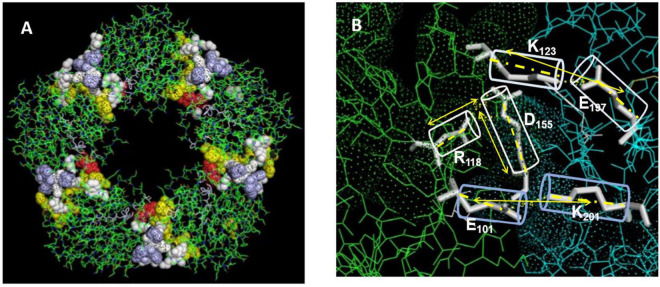
Residues stabilizing pCRP non-covalent inter-subunit contact zones. **(A)** Oriented to show the PC binding recognition face of CRP. Residues Y40TE42 shown as red spheres; Residues P115RVRKSLKK123 shown as yellow spheres; Residues E197VFTKP202 shown as light blue spheres. Residues contributing dominant salt bridges R118-D155; E101-K201; K123-E197 shown at white spheres. **(B)** Displays stick figures and bond distances of stabilizing salt bridges E101-K201: 3.3 Å; K123-E197: 3.4. Å; R118-D155: 3.2 Å. Note the E101-K201 and -K123-E197 salt bridges are linear while the R118-D155 salt bridge occurs at an acute angle. Lv et al. (108) identified the R118-D155 salt bridge as an absolute requirement for the assembly of CRP into its pentameric configuration. Ligands affecting the integrity of this salt bridge would affect the stability of the pentamer, and by extension, the expression of the mCRP, biologically active mCRP conformer.

The CRP pentamer associates non-covalently into its discoid structure using electrostatic, apolar and hydrogen bonding forces. External ligand affecting any of these bonds may influence CRP packing and, by extension, bioactivity. Key electrostatic interactions defined by high-resolution structural analyses identified various salt bridges that contribute to inter-subunit contact stabilization of the pentameric conformation (19, 41). Specific sequences at the five inter-subunit contact sites involve residues in the 114–123 loop (**K**_114_P**R**V**RK**SL**KK**_123_) (containing six closely spaced cationic amino acids (shown in bold type), sequence 187–202 (W_187_**R**AL**K**YEVQGEVFT**K**P_202_) near the C-terminal end of each subunit, and residues 40–42 (Y_40_TE_42_) which localize to the middle of the CBP. Specific residues involved in electrostatic stabilization include **R**_118_ binding to D_155_ on juxtaposed subunits, E_101_ binding to **K**_201_, and E_197_ binding to **K**_123_. Arginine 118 is also localized near the carboxyl group of Proline 202 of the inter-subunit stabilizing sequence described above. The R_118_→D_155_ interaction is sequestered deep in the interaction zone with the carboxyl oxygen of D_155_ approximately 3.4 Å from the guanidino nitrogen in R_118_ (Figure 3). Using iterative programs to predict thermodynamically predicted structures for the relaxed mCRP isoform (42–47), there is a large rotation of these sequence when a pCRP subunit converts into mCRP, substantially increasing the distance between these residues. Any external force that can compete with this intramolecular salt bridge and weaken it (such as a ligand expressing an arginine cationic charge) can promoting ligand-induced conversion of pCRP into mCRP. In appreciation of the significantly different bioactivities of each CRP isoform, this pathway has relevance to how CRP is altered to control its pro- and anti-inflammatory activities (40, 48). The application of expressed arginine residues in both apolipoprotein B (and apolipoprotein E) to CRP binding are discussed below.

**FIGURE 4:**
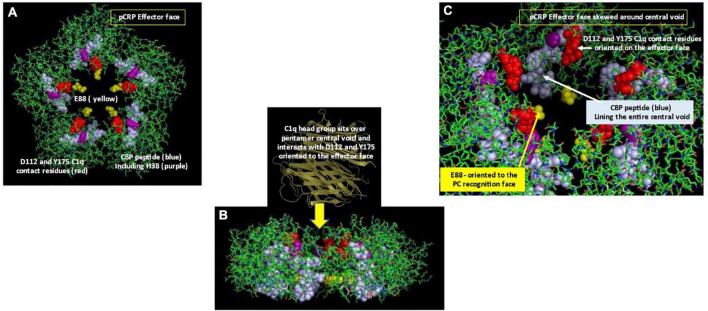
Orientation of C1q head group and Cholesterol Binding Peptide (CBP) residues in pCRP. **(A)** Depicts the Effector (helical) face of the pCRP pentameric disc with a top-down look of pCRP bound to membrane PC ligands. Residues D112 and Y175, which directly react with the C1q globular head group (50) shown in red. The Cholesterol binding peptide is shown in blue. Included within CBP is C1q binding reside H38 (shown in purple). C1q binding residue E88 shown in yellow points into the central void but orients to the PC-recognition face, far removed from the effector face in intact pCRP. **(B)** Depicts a side-view of pCRP shown in panel **(A)**. Initial C1q head group binding occurs symmetrically over all five subunits over the central void on the effector face (16). Note the CBP and E88 residues orient toward the recognition face. To fully expose C1q binding and CBP residues, a conformational change in CRP is required. Binding interactions of CRP with activated membranes including PC groups and apolar regions at the membrane hydrophobic/hydrophilic interface, coupled with forces provided by the large C1q head group contribute energies needed for pCRP conformational change into mCRP. Furthermore, membrane bound cationic groups (e.g., choline, stearyl amine) could provide electrostatic binding to recognition face oriented E88 to help the non-covalently associated CRP subunits to dissociate and structurally rearrange into mCRP. **(C)** Depicts a portion of pCRP as shown in panel **(A)**, skewed to show how direct contact residues D112 And Y175 (red) for C1q binding orient to the effector face, near the central void, being more accessible for initial C1q head group binding. Note the CBP residues (blue) line the entire central void from the effector face to the recognition face. Also, note the orientation of C1q binding residue E88 oriented on the opposite face of the pCRP pentameric disc.

Electrostatic binding residues have also been implicated in controlling CRP binding to the C1q component and to CRP-medicated complement activation (49). Using mutational analyses, the C1q binding site on CRP involves a binding pocket lined with charged residues H_38_, E_88_, and D_112_ (50); CRP residue Y_175_ also provides hydrogen bonding energy in the CRP-C1q interaction (51). D_112_ and Y_175_ directly contact C1q, and, along with H_38_, are critical for complement activation. Li et al. (18) showed CRP’s CBP was predominantly involved in CRP-C1q interactions, with peptides L_83_FEVPEVT_90_ and A_92_PVHICTSWESASGI_106_ contributing energy to the binding reaction. Of note, peptide 92–106 involves the intrachain disulfide bond which is localized two residues removed from H_38_. Access to each of these sites would require conformational rearrangement of CRP. Braig et al. (16) discussed in the CRP-C1q interaction in greater detail, showing how C1q globular head groups could interact on the effector face side of the central void of a partially relaxed CRP pentamer, described as pCRP*. Residues D_112_ and Y_175_ are juxtaposed and are aligned around the central core of pCRP, being most accessible for interaction with the C1q globular head group when CRP is in its compacted pentameric configuration. The CBP peptide is localized on each subunit around the central void nearer the recognition face of pCRP. Exposing these residues for C1q binding would require a structural change in CRP. Using size measurements, the C1q head group is too large to fit inside the void but could provide binding energies to push the loosely associated CRP subunits in pCRP* into the irreversible mCRP conformation, releasing its pro-inflammatory effects (summarized in Figure 4).

Cationic arginine residues on C1q are reported as critical for its interaction with immunoglobulin (52) and CRP (51). Hence, anionic aspartate (D) and glutamate (E) residues on CRP are particularly relevant for arginine-based salt bridges.

## Comparison of apolipoprotein B and apolipoprotein E structures affecting binding reactivities

While apolipoprotein E and apolipoprotein B are distinctive proteins, apo E is known to have binding affinity for the same LDL receptor that binds apo B. Its cationic binding sequence resembles the cationic nonapeptide sequence defined for apo B binding to LDL-R (Table 1).

**TABLE 1:** Comparison of cationic sequences of apo B and apo E involved in interaction with the LDL-receptor.

	**Comparison of Cationic Sequences binding LDL Receptors**									
	**3359**	**3360**	**3361**	**3362**	**3363**	**3364**	**3365**	**3366**	**3367**	
		
Apo B	R	**L**	**T**	**R**	**K**	**R**	**G**	**L**	**K**	apo E3 and E4
Apo E	**R**	**K**	**L**	**R**	**K**	**R**	**L**	**L**	**R**	**C**– > **R**
		
	142	143	144	145	146	147	148	149	150	158

The yellow shading is meant to highlight all cationic residues in these comparable sequences (i.e., R (arginines) and K (lysines).

Apolipoprotein E biofunction involves coordinating the binding of lipoproteins of various sizes and shapes (e.g., LDL, VLDL and HDL) to lipoprotein receptors, especially to LDL-receptor (LDL-R). Comprised of 299 amino acids, the ∼34 kD apo E contains two major functional domains linked by a protease-sensitive hinge peptide. Its N-terminal domain comprises ∼63% of its primary sequence (i.e., residues 1–191) and contains the cationic peptide sequence known to bind LDL receptor. Its C-terminal domain comprises ∼30% of its sequence (i.e., residues 210–299) and contains phospholipid binding residues that anchor apo E to lipoprotein surfaces.

There is a high percentage of basic amino acids in repeated clusters throughout apo E, with arginine accounting for 11% of the amino acid content of the protein. Its N-terminal sequence is divided into hydrophilic 4 helical domains, the fourth of which contains the cationic receptor binding sequence. Its C-terminal domain contains binding residues for polar head groups of phospholipids and for apolar residues to better anchor apo E to the hydrophobic/hydrophilic interface of a lipoprotein particle. Apo E binds to the polar head groups of lipid molecules and extends over the lipoprotein surface rather than inserting into the hydrophobic lipid core of the particle. This orientation facilitates HDL core expansion with cholesterol esters, in a way that is not limited by a requirement for apolar molecular interactions and surface curvature issues inherent in differed sized and shaped HDL particles. De-lipidated apo E does self-aggregate into oligomers. However, mutating selective residues in the C-terminal domain can abrogate self-aggregation allowing for the detailed study of monomeric apo E proteins (53–59).

**FIGURE 5:**
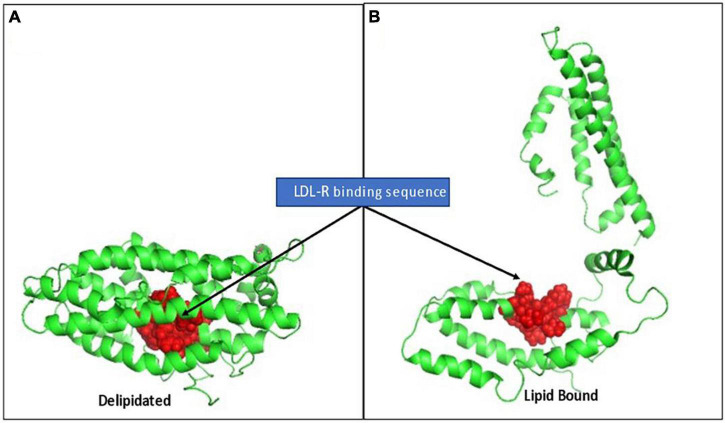
Rendition of the structural change in apo E elicited by lipid binding, exposing the LDL-R binding sequence. (A) Depicts full length apo E3 (PBD: 2L7b) structural packing as a delipidated protein. The cationic nonapeptide mediating apo E binding to LDL-receptors (R142KLRKRLLR150) shown as red spheres, being inaccessible in the interior of the compacted lipid-free tertiary structure. (B) Based on Chen et al. (53), who described a two-step conformational change in apo E on lipid binding, this rendition is provided to visualize how lipid binding can contribute to apo E conformational changes that alter access to its receptor binding sequence and, in turn, its biofunction in mobilizing lipoprotein/membrane associated cholesterol.

The strongly cationic nonapeptide sequence mediating apo E’s binding to the LDL-receptor (R_142_KLRKRLLR_150_) is buried and inaccessible in delipidated apo E. In the absence of lipids, apo E collapses upon itself forming a globular tertiary structure in which its N-terminal and C-terminal domains are stabilized by five salt bridges, hydrogen bonds and apolar interactions. The cationic LDL-R binding sequence is hidden in this compacted structural orientation. Exposing this binding sequence follows a two-step process involving a marked conformational change in the tertiary structure of apo E. Step 1 is rapid and involves phospholipid binding or the C-terminal domain to exposed phospholipid head groups on lipoprotein particles. Apo E sits on the polar head groups (e.g., phosphocholine) of these phospholipids, being juxtaposed to but not inserted into the apolar membrane region. As apo E associates with lipids, the salt bridges holding its two domains together weaken, relaxing the tertiary structure of the apoprotein. Step 2 is a slower, reversible process involving relaxing the N-terminal domain into an open conformation with increased exposure of the LDL-R binding peptide (53, 60). To provide perspective on the major conformational rearrangement of apo E in the absence and presence of lipids, Chen noted the distance between residues N-terminal domain residue C_112_ and C-terminal domain residue W_264_ (specifically for apo E3) was ∼28Å in the absence of lipid, but > 80 Å in its presence (see Figure 5 for perspective of these structural changes).

Apo E is known to exist in three allelic forms which involve specific cysteine to arginine mutations at 2 sites in its 299 amino acid sequence, one of which is C_112_ mentioned above. Apo E2 contains cysteine residues at both position 112 and 158 (i.e., C_112_, C_158_). Apo E3 contains a cysteine residue at position 112, but an arginine residue at position 158 (i.e., C_112_, R_158_). Apo E4 contains arginine residues at both positions 112 and 158 (i.e., R_112_, R_158_). In apo E4, the arginine residue expressed at position 112 adds an additional salt bridge to residue E_109_ within the N-terminal domain, which also causes a structural rotation that increases exposure of R_61_. R_61_ becomes accessible to form the additional salt bridge with C-terminal domain residue E_255_ (54). Mahley went on to report (56) that the additional salt bridge in apo E4 is of importance in the increased neuro-degenerative pathologies associated with this isoform.

Each expressed protein differentially binds lipids and apo E receptors, including the LDL receptor. The binding affinity of apo E4 to LDL-R was stronger than apo E3, which in turn was stronger than apo E2 (61). As the mutated residues are in the N-terminal aqueous, receptor binding domains of apo E and not the C-terminal lipid-binding domain, apo E4 shows stronger binding to the LDL-R but less capacity to process cholesterol than other apo E alleles (57, 59). As the brain contains 23% of total body cholesterol (62), processes that affect cholesterol transport in the brain can have pathophysiological consequences.

Apo E also binds (anionic) heparin sulfate proteoglycans and amyloid-beta (Aβ) peptides. Heparin binding, presumably by competing for intramolecular salt bridges, can open up apo E structure to allow for LDL-R binding. Different apo E isotypes can affect how αβ peptides are generated from the transmembrane amyloid precursor protein (APP) and how they are aggregated and cleared (63). The toxicity associated with αβ peptide deposition is associated with hyperphosphorylation of the microtubular protein tau which can lead to neurofibrillary tangles in the brains of Alzheimer’s patients (53, 54, 58).

## Bioeffects of C-reactive protein-lipid/apolipoprotein complexes

CRP admixed with liposomes or Large Unilamellar Vesicles (LUVETs) has anti-cancer activity both *in vitro* and *in vivo* [summarized in Potempa et al. (48)].

Bian et al. (64) reported that the interaction of CRP could increase movement of apo B-containing LDLs across endothelial cells and promote development of atherosclerotic plaques. The effect of CRP required NAPDH oxidase to generate ROS and was blocked when sulfhydryl groups were reduced.

Li et al. (65) investigated how CRP could affect LDL directional transport across endothelial cell barriers. Transport occurred both into and out of tissues with a preference for movement from blood into tissues. The directional movement was regulated by the basement membrane matrix such that, when the basement membrane was altered, CRP was redirected back into the blood.

In rat studies, peritoneal macrophages differentially processed CRP-LDL complexes compared to CRP-acetylated-LDL (i.e., altered) complexes (66). Altered LDL particles bound a scavenger receptor as opposed to normal LDL binding to LDL receptors. Shih et al. (67) later reported CRP mediated altered LDL binding more specifically to the LOX-1 scavenger receptor on endothelial cells, and that arginine residues on the receptor were involved in this interaction. In direct macrophage binding and uptake studies of pCRP and mCRP isoforms with both normal LDL and oxidized LDL particles, the mCRP isoform and not the pCRP isoform enhanced binding and uptake of normal LDLs to macrophage LDL receptors but reduced binding and uptake of oxidized LDL (12, 68).

In mouse models of atherosclerosis using both transgenic models expressing human CRP, or knockout models of endogenous CRP, CRP has been differently reported to (1) accelerate progression of atherosclerosis in apo E-deficient mice (69); (2) slow the development of atherosclerosis (70, 71); and (3) have no effect (72). These reports were completed before there was substantial literature describing distinctive CRP isoforms and did not consider how each of pCRP and mCRP may have influenced results generated. As studies evolved, it is now clearly apparent that different CRP isoform do have different pro- and anti-atherogenic effects. Schwedler et al. (73) used an apo E knockout mouse model showed pCRP injections increased aortic plaque size while modified CRP decreased plaque formation. The observed effects of mCRP were related to regulation of reactive oxygen and peroxynitrite formation (i.e., pro-inflammatory stimulation) but in ways that impaired vascular relaxation responses (74). Ji et al. (12) and Schwedler et al. (75) showed mCRP but not pCRP preferentially associated with oxidized LDL. As mentioned above, mCRP inhibited altered LDL uptake by macrophages in a way that reduced formation of atherogenic plaques. The mCRP effect did not involve Fcγ receptors CD16 (which binds mCRP) nor CD32 (which binds pCRP) [summarized in Wu et al. (15)] nor the LOX-1 scavenger receptor on macrophages (as opposed to endothelial cells).

Liposome-bound CRP activates complement. Effects require liposomes to contain either phosphatidylcholine (PtC) or sphingomyelin (both PC containing lipids). CRP-mediated complement effects were influenced by liposome lipid acyl chain length, degree of unsaturation, and cholesterol content (76). The addition of a positive charge either as stearyl amine or cetyltrimethylammonium bromide, or addition of galactosyl ceramide to certain liposomes, improved CRP binding and complement activation activities.

CRP complexed with lipoproteins can also activate complement (77, 78). Binding to lipoproteins requires CRP’s primary binding ligand, phosphocholine, be made accessible by either enzymatically treating or oxidizing LDLs (79, 80). Wang et al. (81) showed how lipoprotein oxidation affects surface curvature and promotes CRP binding. The average diameter of LDL particles is 25–26 nm, but oxidation decreases particle size and increases surface curvature. CRP preferentially bound to highly curved, smaller lipoprotein mimetic particles. When bound by these smaller, highly curved particles, pCRP not only binds but is structurally altered into the mCRP isoform, which stays associated with the lipoprotein particle (resisting dissociation with chelators or disruptive washing treatments). To specifically look at how each of the pCRP and mCRP isoforms affected complement activation pathways, Ji et al. (12) showed mCRP rather than pCRP could bind the C1q component of the classical C system through its collagen-like stem. Lipoprotein bound mCRP activated the classical C pathway, but also recruited the alternative pathway regulating protein Factor H to the CRP-activated surface. Factor H functions to limit the production of the membrane attack complex (MAC) suggesting conformationally altered CRP, after insertion into lipid membranes, can promote Complement-mediated opsonization but inhibit C-mediated cell lysis pathways.

In addition to complement, CRP-lipoprotein particles regulate coagulation pathways involving tissue factor and thrombin activation. Using Phosphatidyl serine/Phosphatidyl choline (PtS: PtC) liposomal vesicles as an anchor for tissue factor, CRP inhibited factor VIIa mediated tissue factor activation of Factor X and the initiation of fibrin formation (82, 83). The capacity of CRP to combine with VLDL was also linked to processes that contribute to disseminated intravascular coagulation (DIC) (83, 84). Also, CRP has been implicated in fat embolism pathologies associated with therapeutic infusions of lipid emulsions (i.e., “Intralipid”) (85, 86). Even without intralipid infusion, extremely high CRP blood levels (>200 μg/ml) appear to complex with VLDLs in blood and reduced normal phagocytic cell function, which in turn affects the clearance of bacteria in models of sepsis (87). Very high blood CRP levels are known to be associated with poor outcomes to any disease (48, 88). Its association with VLDLs suggests a mechanism by which CRP can contribute to morbidity. Table 2 summarizes distinctive bioactivities of pCRP and mCRP with a focus on lipid and lipoprotein.

**TABLE 2:** Key observations of pCRP and mCRP interactions with lipids and lipoproteins.

	Pentameric CRP (pCRP)	Monomeric, modified CRP (mCRP)
Binding to lipids	• pCRP binds exposed phosphocholine (PC) groups of the phospholipids of membranes, liposomes, and lipoproteins as a function of calcium • Key factors promoting pCRP binding include lipid acyl chain length (shorter chains), degree of unsaturation and cholesterol content • Also, including of a positive charge as either as stearyl amine or cetyltrimethylammonium bromide improved binding • pCRP calcium-dependent binding was also seen when Phosphoethanolamine (PE) was used instead of phosphocholine (PC)	• mCRP does not express calcium dependent PC binding specificity • Monolayer technique experiments established mCRP interacts with the hydrophobic tail of LDL lipids through membrane insertion • In the presence of calcium, mCRP induced substantial increase in monolayer membrane pressure formed by LDL lipid extracts; indicates membrane insertion • mCRP interacts with RBC ghost membranes and is highly resistant to alkaline carbonate and high salt extraction; indicates it inserts into bilayers like an integral protein • The majority of RBC ghost-associated mCRP remained insoluble with Triton X-100; this is consistent with the behavior of lipid raft-resident proteins

Complement activation	• The positive charge or presence of galactosyl ceramide to certain liposomes improves pCRP binding and complement activation activities • CRP binds C1q of the classical pathway on the opposite face of the pentameric disc from the PC-binding face	• mCRP binds C4bp and enhances degradation of C4b and C3b • C1q and C4bp compete for mCRP binding • mCRP binds factor H and factor H-like protein 1 (FHL1) and modulates the alternative complement pathway

Binding to Apolipoproteins	• pCRP binds apo B; binding does not require calcium	• mCRP binds apo B at a greater affinity that pCRP • mCRP binds both isolated apo B and LDL-associated apoB through its cationic peptide sequence • mCRP binding to native LDL can be inhibited by competitors that interfere with electrostatic interactions and apolar interactions; indicates mCRP interacts with lipoproteins using electrostatic binding and apolar binding

Effect of modification of lipoproteins	• pCRP did not bind native LDLs either in the presence of Calcium or EDTA • Lipoproteins oxidized to alter fatty acyl chain length and unsaturation do bind pCRP through calcium-dependent interactions with exposed PC groups • Lipoproteins enzymatically treated to cleave apolipoproteins bound pCRP as a function of calcium	• Once bound to native LDL, mCRP is not eluted by high salt indicating a binding is primarily apolar • mCRP interacted with lipid extracts from each of native, oxidized and enzymatically treated lipoproteins

Observations relevant to unrecognized pCRP conversion to mCRP	• Binding of “CRP” to apo B/lipoproteins best when CRP was aggregate and immobilized. • Aggregated CRP immobilized on a surface (conditions shown to from the mCRP isoform) bound LDL and VLDL from normal human serum (i.e., apoB-containing lipids). • Various experimental results with strongly chelated pCRP are more like results generated with mCRP than pCRP in calcium • pCRP in calcium will bind to exposed PC groups in membrane surfaces but initially, will not insert into the apolar lipid zone. With prolonged incubation times (e.g., 2–4 h) membrane associated CRP will insert into hydrophobic zones	

Relevant references: (4, 37–40, 107, 109–112).

## C-reactive protein and extracellular vesicles/microparticles

Extracellular lipid vesicles released from cell membranes or synthesized to carry various biochemical molecules (e.g., mRNA-based vaccines) represent an emerging field of diagnostic and therapeutic medicine (89). Of relevance to this review, the mCRP isoform has been shown to associate with small, lipid microparticles found in circulation.

mCRP forms on and inserts into membranes to as a regulatory step in eliciting the biofunction of CRP as an acute phase reactant. As a strong pro-inflammatory mediator, mCRP will activate leukocytes to stimulate and amplify the inflammatory response, producing ROS and secreting enzymes that affect the tissue environment, including the membrane into which mCRP inserted. The destructive power of leukocyte activation creates bits of sloughed membranes, which can carry mCRP and enter circulation as extracellular lipidic vesicles. Crawford et al. (90) showed patients with peripheral artery disease (PAD) did express mCRP-associated microparticles in blood. These particles, ranging in size from 0.1 to 1 μm [compared to platelets (3 μm), RBCs (7 μm) lymphocytes (7–10 μm) and polymorphonuclear leukocytes (PMNs) (15–25 μm)], and were primarily derived from activated endothelial cells (verified using co-marker FACS analyses). While these microparticles are derived from plasma membrane lipids, their small size and high curvature results in PS and PE lipids, normally found on the inner leaflet of an intact plasma membrane, found in high concentration on the surface of microparticles.

Habersberger et al. (91) showed mCRP-lipid microparticles were elevated in blood of patients after a myocardial infarction. These microparticles were enriched in lyso-PC (monoacyl phosphatidyl choline) and were shown to enhance the conversion of pCRP into mCRP. Isolated mCRP-associated microparticles did have pro-inflammatory bioactivity when added to endothelial cells in culture.

Habersberger’s group also showed that formation of mCRP from pCRP on a membrane surface could be prevented if pCRP was first neutralized by a bivalent bis-PC compound that links two juxtaposed pentamers (92). This compound binds two PC binding sites in a way that forms a recognition face-to-recognition face soluble decamers which can no longer bind membrane exposed PC groups. As pCRP is not localized to an apolar membranous zone, insufficient biochemical energy is available to loosen the pentameric structure and lead to expression of the mCRP isoform.

Trial et al. (5) discussed how lipid microparticles can bind both pCRP and mCRP. Adding pCRP to freshly isolated microparticles from cardiac patient blood resulted in conversion of pCRP to mCRP within 20 minutes. Of note, lipid-associated CRP antigens could not be quantified using standard CRP nephelometric measurement assays. Furthermore, the level of FACS-quantified mCRP-lipid complexes did not correlate with lipid-free pCRP concentration in blood, even at high sensitivity levels (i.e., 1–10 μg/ml).

Taken together, while highly soluble pCRP can exist as a lipid-free protein in blood, binding to an activated membrane surface through PC or cationic ligands, can localize CRP to apolar biochemical energies that contribute to its dissociation and structural change into mCRP. Structurally altered mCRP expresses a cholesterol binding domain and enters lipid rafts which activate and amplify pro-inflammatory signaling pathways of the acute phase of an inflammatory response. Activated leukocyte effector responses at involved tissue sites will enzymatically degrade mCRP or cause it to be sloughed away from activated cell surface as a lipid microparticle complex, resulting in down-regulation of the acute inflammatory response. Serum soluble pCRP thus circulates as a pro-activator of inflammation, requiring interaction with lipids to release its important bioactivities in host defense responses.

## Clinical relevance of C-reactive protein-lipoprotein interactions

CRP-lipid interaction in atherosclerotic disease has been a focus topic of many studies. Before the understanding that CRP exists in at least two distinctive isoforms, diametrically opposite conclusions were reached for directly comparable experimental model systems. When reagents were developed to differentially study pCRP from mCRP, results consistently showed pCRP prevented, while mCRP promoted monocyte processing of potentially atherogenic LDL particles (68). Even though mCRP is a strong pro-inflammatory mediator, it lessened atherogenesis of modified LDLs in animal models of disease (93). Further, the CRP effect on atherosclerosis did not depend on PC binding as, when mutant proteins were constructed to lose PC binding activity (i.e., F_66_A/T_76_Y/E_81_A), “CRP” still bound atherogenic LDLs and, when injected into atherogenic-prone mice, slowed disease progression, and reduced the size of aortic lesions (94). Recently, Cheng et al. (95) showed the dose level of injected mCRP was an important factor in protecting from induced liver disease. At lower doses, mCRP conferred protection from disease, but at higher doses, this protection was lost, attributed to mCRP ability to over-stimulate the *in situ* inflammatory response.

In the Schwedler et al. (73) study using an apo E knockout mouse model, pCRP injections increased aortic plaque size while modified CRP decreased plaque formation. Histologically, the mCRP antigen co-localized with deposits of apolipoprotein B and macrophages. The observed effects of mCRP were related to regulation of ROS and RNS formation and vascular relaxation responses, underscoring that mCRP’s effects in stimulating acute inflammatory responses may have positive therapeutic benefits (48, 74, 75).

Other studies suggest a pathological role for mCRP in atherosclerosis, thrombosis, angiogenesis, and cerebrovascular pathologies (96, 97). mCRP antigen has been found in brain tissues associated with damaged micro vessels and in human and mouse brains involved with neuroinflammation (98–100). Since mCRP forms from pCRP, drugs that inhibit the *in situ* formation of mCRP from pCRP could have therapeutic value in treating both systemic and cerebral inflammatory diseases (3, 101).

Alzheimer’s disease involves neuroinflammation and dysfunction in biochemical processing of cholesterol in the brain (102, 103). Over the past decade, individuals expressing the apo E4 allele have been shown to have increased risk for developing early onset Alzheimer’s disease and appear to have poorer outcomes following any type of severe brain injury. A better understanding of the relevance of apo E proteins to inflammatory processes and to cholesterol balance will contribute to medical advances in the prevention and treatment of this disease. A relationship between apo E4 and plasma CRP levels relevant to the development of Alzheimer’s disease has been reported (104). Furthermore, the mCRP isoform bound to endothelial cell CD31 (a receptor mediating platelet and leukocyte binding and transcytosis), influencing apo E4-related responses in the development of Alzheimer’s disease (100).

## Summary

CRP has been known since the 1940s as a diagnostic marker for inflammation. Its blood levels change rapidly and pronouncedly with any tissue damaging process that involves non-memory, innate immune defense system activation, leading to its designation as the “prototypic acute phase reactant” of host defense responses. Its exact biological role as a key protein in this process has been an area of uncertainty and confusion covering decades of detailed study.

While most detailed studies of CRP structure/function relationships have focused on the lipid-free, highly aqueously soluble, non-covalently linked, non-glycosylated pentameric protein, substantial literature exists that describes CRP as a blood protein that also associates with apolipoprotein B-expressing lipoproteins. While a primary binding reactivity with lipoproteins involves calcium-regulated binding affinity with exposed phosphocholine groups, CRP also directly binds to a cationic peptide sequence as expressed on the apolipoprotein. In either case, bound CRP is localized to and interacts with lipid surfaces (i.e., lipoproteins or membranes). Little attention has been given to the role and influence of amphipathic lipid molecules as regulators of CRP structures and bioactivities.

A key evolution in understanding CRP’s role as a biological response modifier was the recognition that when dissociated, CRP subunits undergo a rapid, irreversible conformational rearrangement into an isoform with strong affinity for apolar regions of lipid surfaces. When CRP is brought into juxtaposition with an apolar zone, the localized non-polar biochemical energies not only help dissociate the pentamer and contribute to the conversion of CRP into the mCRP isoform, but a novel binding site for membrane-bound cholesterol is expressed. As mCRP is formed, it is drawn into a membrane where it stimulates activation and signaling pathways that contribute to a strong pro-inflammatory host defense responses (which more accurately describes “CRP” as the prototypic acute phase reactant) (105). Conformational rearrangement of CRP from the pentamer to the modified monomer results in significate loss of aqueous solubility, loss of antigenicity associated with the pentamer, and expression of new (neo) epitopes associated with the conformational isomer. Once formed, mCRP sequesters into lipid zones which masks its detection using assays and reagents developed for the non-lipid-associated highly aqueously soluble protein found in blood and body fluids.

Lipid-associated mCRP can be sloughed off activate membrane surfaces into body fluids, being found associated with micro-particles. The strong lipid association is of relevance to reassessing all prior studies describing CRP lipoprotein associations in blood. As lipid-soluble mCRP can be formed from pCRP, which initially binds to the aqueous surface of lipoproteins using its calcium-regulated affinity for PC groups, any CRP that is not readily dissociated from the lipoprotein particle by simple chelation, must be evaluated as the lipid bound mCRP isoform rather than the pCRP isoform. As pCRP and mCRP are now known to have distinctive anti- and pro-inflammatory bioactivities, respectively, it is possible to reassess the role(s) CRP may play in different lipoprotein-involved pathophysiologies such as cardiovascular diseases and neurodegenerative diseases.

## Author contributions

LP researched, organized, and wrote the manuscript. WQ and AS reviewed and edited the manuscript for clinical accuracy and clarity. IR researched, edited, verified references, contributed to the figures, and validated biochemical and analytical concepts included. All authors contributed to the article and approved the submitted version.

## Abbreviations

pCRP: pentameric C-reactive protein
mCRP: monomeric, modified C-reactive protein
VLDL: very low density lipoproteins
LDL: low density lipoproteins
LDL-R: LDL receptor
apo B: apolipoprotein B
apo E: apolipoprotein E
CPS: C-polysaccharide fraction of pneumococcal cell walls
PC: phosphocholine
PtC: phosphatidyl choline
PtS: phosphatidyl serine
DEAE: diethylaminoethyl
GuHCl: guanidinium hydrochloride
mCRP_*m*_: membrane-associated modified CRP (an intermediate structure retaining pentameric orientation while also expressing mCRP-antigenicity)
pCRP*: a partially changed pentameric CRP structure that expresses some mCRP-specific properties
CBP: cholesterol binding peptide sequence of CRP.

## References

[1] Volanakis JE Clements WL Schrohenloher RE. C-reactive protein: purification by affinity chromatography and physicochemical characterization. *J Immuno Meth.* (1978) 23:285–95. 10.1016/0022-1759(78)90203-X

[2] Volanakis JE Wirtz KW. Interaction of C-reactive protein with artificial phosphatidylcholine bilayers. *Nature.* (1979) 281:155–7. 10.1038/281155a0 471064

[3] Caprio V Badimon L Di Napoli M Fang WH Ferris GR Guo B pCRP-mCRP dissociation mechanisms as potential targets for the development of small-molecule anti-inflammatory chemotherapeutics. *Front Immunol.* (2018) 9:1089–96. 10.3389/fimmu.2018.01089 29892284 PMC5985513

[4] Rajab IM Majerczyk D Olson ME Addams JMB Choe ML Nelson MS C-reactive protein in gallbladder diseases – diagnostic and therapeutic insights. *Biophys Rep.* (2020) 6:49–67. 10.1007/s41048-020-00108-9

[5] Trial J Potempa LA Entman ML. The role of C-reactive protein in innate and acquired inflammation: new perspectives. *Inflammation Cell Signal.* (2016) 3:e1409–18. 10.14800/ics.1409 PMC5058362 27738646

[6] Heegaard NH Robey FA. A capillary electrophoresis-based assay for the binding of Ca2+ and phosphorylcholine to human C-reactive protein. *J Immunol Methods.* (1993) 166:103–10. 10.1016/0022-1759(93)90333-3 8228279

[7] Christopeit T Gossas T Danielson UH. Characterization of Ca2+ and phosphocholine interactions with C-reactive protein using a surface plasmon resonance biosensor. *Anal Biochem.* (2009) 391:39–44. 10.1016/j.ab.2009.04.037 19435596

[8] Mikolajek H Kolstoe SE Pye VE Mangione P Pepys MB Wood SP. Structural basis of ligand specificity in the human pentraxins, C-reactive protein and serum amyloid P component. *J Mol Recognit.* (2011) 24:371–7. 10.1002/jmr.1090 21360619

[9] Zeller J Shing KC Nero T Krippner G McFadyen J Bogner B Kreuzaler S *Discovery, in-vitro, and in-vivo efficacy of an anti-inflammatory small molecule inhibitor of C-reactive protein. Research Square preliminary report.* (2021) Available online at: 10.21203/rs.3.rs-944388/v1

[10] Gotschlich EC Edelman GM. Binding properties and specificity of C-reactive protein. *Proc Natl Acad Sci USA.* (1967) 57:706–12. 10.1073/pnas.57.3.706 16591521 PMC335566

[11] Robey FA Jones KD Tanaka T Liu T-Y. Binding of C-reactive protein to chromatin and nucleosome core particles. *J Biol Chem.* (1984) 259:7311–6. 6427230

[12] Ji SR Wu Y Potempa LA Qiu Q Zhao J. The interactions of low-density lipoprotein with different forms of C-reactive protein: implication of an active role of modified C-reactive protein in the pathogenesis of atherosclerosis. *Int J Biochem Cell Biol.* (2006) 38:648–61. 10.1016/j.biocel.2005.11.004 16376133

[13] Ji SR Wu Y Zhu L Potempa LA Sheng FL Wei L Cell membranes and liposomes dissociate C-reactive protein (CRP) to form a new, biologically active structural intermediate: mCRPm. *FASEB J.* (2007) 21:284–94. 10.1096/fj.06-6722com 17116742

[14] Ji S-R Bai L Shi J-M Li H-Y Potempa LA Filep JG Monomeric C-reactive protein activates endothelial cells via interaction with lipid raft membrane microdomains. *FASEB J.* (2009) 23:1806–16. 10.1096/fj.08-116962 19136614

[15] Wu Y Potempa LA El Kebir D Filep JG. C-reactive protein and inflammation: conformational changes affect function. *Biol Chem.* (2015) 396:1181–97. 10.1515/hsz-2015-0149 26040008

[16] Braig D Nero TL Koch H-G Kaiser B Wang X Thiele JR Characterization of transitional changes in the CRP structure leading to the exposure of pro-inflammatory binding sites. *Nat Commun.* (2017) 23:14188–207. 10.1038/ncomms14188 28112148 PMC5264208

[17] Wang M-Y Ji S-R Bai C-J El Kebir D Li H-Y Shi J-M A redox switch in C-reactive protein modulates activation of endothelial cells. *FASEB J.* (2011) 25:3186–96. 10.1096/fj.11-182741 21670067

[18] Li HY Wang J Meng F Jia ZK Su Y Bai QF An intrinsically disordered motif mediates diverse actions of monomeric C-reactive protein. *J Biol Chem.* (2016) 291:8795–804. 10.1074/jbc.M115.695023 26907682 PMC4861447

[19] Shrive AK Cheetham GM Holden D Myles DA Turnell WG Volanakis JE Three dimensional structure of human C-reactive protein. *Nat Struct Biol.* (1996) 3:346–54. 10.1038/nsb0496-346 8599761

[20] Mackiewicz MR Hodges HL Reed SM. C-reactive protein induced rearrangement of phosphatidylcholine on nanoparticle mimics of lipoprotein particles. *J Phys Chem B.* (2010) 114:5556–62. 10.1021/jp911617q 20364851 PMC2930195

[21] Ying S-C Shephard E deBeer FC Siegel JN Harris D Gewurz BE Localization of sequence-determined neo-epitopes and neutrophil digestion fragments of C-reactive protein utilizing monoclonal antibodies and synthetic peptides. *Mol Immunol.* (1992) 29:677–87. 10.1016/0161-5890(92)90205-c 1374844

[22] Kushner I Feldman G. Control of the acute phase response. Demonstration of C-reactive protein synthesis and secretion by during acute inflammation in the rabbit. *J Exp Med.* (1978) 148:466–77. 10.1084/jem.148.2.466 702046 PMC2184945

[23] Saxena U Nagpurkar A Dolphin PJ Mookerjea S. A study on the selective binding of apoprotein B- and E-containing human plasma lipoproteins to immobilized rat serum phosphorylcholine-binding protein. *J Biol Chem.* (1987) 262:3011–6. 3102482

[24] Saxena U Francis-Collins J Hall J Legal Y Barrowman J Nagpurkar A Removal of apoprotein-B-containing lipoproteins by plasmapheresis using immobilized phosphorylcholine-binding protein affinity adsorbent. *Biochem Cell Biol.* (1990) 68:255–9. 10.1139/o90-035 2350492

[25] Schwalbe RA Coe JE Nelsestuen GL. Association of rat C-reactive protein and other pentraxins with rat lipoproteins containing apolipoproteins E and A1. *Biochemistry.* (1995) 34:10432–9. 10.1021/bi00033a015 7544614

[26] Cladaras C Hadzopoulou-Cladaras M Nolte RT Atkinson D Zannis VI. The complete sequence and structural analysis of human apolipoprotein B-100: relationship between apoB-100 and apoB-48 forms. *EMBO J.* (1986) 5:3495–507. 10.1002/j.1460-2075.1986.tb04675.x 3030729 PMC1167386

[27] Cabana VG Gewurz H Siegel JN. Interaction of very low-density lipoproteins (VLDLs with rabbit CRP. *J Immunol.* (1982) 128:2342–8. 6801137

[28] Cabana VG Siegel JN Sabesin SM. Effects of the acute phase response on the concentration and density distribution of plasma lipids and apolipoproteins. *J Lipid Res.* (1989) 30:39–49. 2493057

[29] de Beer FC Soutar AK Baltz ML Trayner IM Feinstein A Pepys MB. Low density lipoprotein and very low-density lipoprotein are selectively bound by aggregated C-reactive protein. *J Exp Med.* (1982) 156:230–42. 10.1084/jem.156.1.230 7086355 PMC2186728

[30] Rowe IF Soutar AK Trayner IM Baltz ML de Beer FC Walker L Rabbit and rat C-reactive proteins bind apolipoprotein B-containing lipoproteins. *J Exp Med.* (1984) 159:604–16. 10.1084/jem.159.2.604 6693835 PMC2187229

[31] Rowe IF Soutar AK Trayner IM Thompson GR Pepys MB. Circulating human C-reactive protein binds very low-density lipoproteins. *Clin Exp Immunol.* (1984) 58:237–44. 6478650 PMC1576975

[32] Dougherty TJ Gewurz H Siegel JN. Preferential binding and aggregation of rabbit C-reactive protein with arginine-rich proteins. *Mol Immunol.* (1991) 28:1113–20. 10.1016/0161-5890(91)90026-g 1922103

[33] Dicamelli R Potempa LA Siegel J Suyehira L Petras K Gewurz H. Binding reactivity of C-reactive protein for polycations. *J Immunol.* (1980) 125:1933–8. 6776184

[34] Potempa LA Siegel J Gewurz H. Binding reactivity of C-reactive protein for polycations. II Modulatory effects of calcium and phosphocholine. *J Immunol.* (1981) 127:1509–14. 7276568

[35] Siegel J Rent R Gewurz H. Interactions of C-reactive protein with the complement system I. Protamine-induced consumption of complement in acute phase sera. *J Exp Med.* (1974) 140:631–47. 10.1084/jem.140.3.631 4472155 PMC2139624

[36] Siegel J Osmand AP Wilson MF Gewurz H. Interactions of C-reactive protein with the complement system II. C-reactive protein-mediated consumption of complement by poly-L-lysine polymers and other polycations. *J Exp Med.* (1975) 142:709–21. 10.1084/jem.142.3.709 809531 PMC2189923

[37] Mold C Rodgers CP Richards RL Alving CR Gewurz H. Interaction of C-reactive protein with liposomes. III. Membrane requirements for binding. *J Immunol.* (1981) 126:856–60. 7462634

[38] Richards RL Gewurz H Siegel J Alving CR. Interactions of C-reactive protein and complement with liposomes. II. Influence of membrane composition. *J Immunol.* (1979) 122:1185–9. 448084

[39] Mihlan M Blom AM Kupreishvili K Lauer N Stelzner K Bergstrom F Monomeric C-reactive protein modulates classic complement activation on necrotic cells. *FASEB J.* (2011) 25:4198–210. 10.1096/fj.11-186460 21856781

[40] Rajab IM Hart PC Potempa LA. How C-reactive protein structural isoforms with distinctive bioactivities affect disease progression. *Front Immunol.* (2020) 112:2126. 10.3389/fimmu.2020.02126 33013897 PMC7511658

[41] Thompson D Pepys MB Wood SP. The physiological structure of human C-reactive protein and its complex with phosphorylcholine. *Structure.* (1999) 7:169–77. 10.1016/S0969-2126(99)80023-9 10368284

[42] Xu J. Distance-based protein folding powered by deep learning. *Proc Natl Acad Sci USA.* (2019) 116:16856–65. 10.1073/pnas.1821309116 31399549 PMC6708335

[43] Xu J Mcpartlon M Li J. Improved protein structure prediction by deep learning irrespective of co-evolution information. *BioRxiv* [Preprint] (2020): 10.1101/2020.10.12.336859 PMC8340610 34368623

[44] Xu J Wang S. Analysis of distance-based protein structure prediction by deep learning in CASP13. *Proteins.* (2019) 87:1069–81. 10.1002/prot.25810 31471916

[45] Wang S Li Z Yu Y Xu J. Folding membrane proteins by deep transfer learning. *Cell Syst.* (2017) 5: 202–211.e3. 10.1016/j.cels.2017.09.001 28957654 PMC5637520

[46] Wang S Sun S Li Z Zhang R Xu J. Accurate de novo prediction of protein tact map by ultra-deep learning model. *PLoS Comput Biol.* (2017) 13: e1005324. 10.1371/journal.pcbi.1005324 28056090 PMC5249242

[47] Wang S Sun S Xu J. Analysis of deep learning methods for blind protein contact prediction in CASP12. *Proteins.* (2018) 86 Suppl 1:67–77. 10.1002/prot.25377 28845538 PMC5871922

[48] Potempa LA Rajab IM Olson ME Hart PC. C-reactive protein and cancer. Interpreting the differential bioactivities of its pentameric (pCRP) and monomeric, modified (mCRP) isoforms. *Front Immunol.* (2021) 12:744129. 10.3389/fimmu.2021.744129 34552600 PMC8450391

[49] Singh SK Ngwa DN Agrawal A. Complement activation by C-reactive protein is critical for protection of mice against pneumococcal infection. *Front Immunol.* (2020) 11:1812. 10.3389/fimmu.2020.01812 32903624 PMC7438579

[50] Agrawal A Shrive AK Greenhough TJ Volanakis JE. Topology and structure of the C1q-binding site on C-reactive Protein. *J Immunol.* (2001) 166:3998–4004. 11238646 10.4049/jimmunol.166.6.3998

[51] Roumenina LT Ruseva MM Zlatarova A Ghai R Kolev M Olova N Interaction of C1q with IgG1, C-reactive protein and pentraxin 3: mutational studies using recombinant globular head modules of human C1q A, B, and C chains. *Biochemistry.* (2006) 45:4093–104. 10.1021/bi052646f 16566583 PMC3874390

[52] Kojouharova MS Gadjeva MG Tsacheva IG Zlatarova A Roumenina LT Tchorbadjieva MI Mutational analyses of the recombinant globular regions of human C1q A, B, and C chains suggest an essential role for arginine and histidine residues in the C1q-IgG interaction. *J Immunol.* (2004) 172:4351–8. 10.4049/jimmunol.172.7.4351 15034050

[53] Chen J Li Q Wang J. Topology of human apolipoprotein E3 uniquely regulates its diverse biological functions. *PNAS.* (2011) 108:14813–18. 10.1073/pnas.1106420108 21873229 PMC3169138

[54] Mahley RW Rall SC Jr. Apolipoprotein E: far more than a lipid transport protein. *Annu Rev Genomics Hum Genet.* (2000) 1:507–37. 10.1146/annurev.genom.1.1.507 11701639

[55] Mahley RW Weisgraber KH Huang Y. Apolipoprotein E4: a causative factor and therapeutic target in neuropathology, including Alzheimer’s disease. *Proc Natl Acad Sci USA.* (2006) 103:5644–51. 10.1073/pnas.0600549103 16567625 PMC1414631

[56] Mahley RW Weisgraber KH Huang Y. Apolipoprotein E: structure determines function, from atherosclerosis to Alzheimer’s disease to AIDS. *J Lipid Res.* (2009) 50:S183–8. 10.1194/jlr.R800069-JLR200 19106071 PMC2674716

[57] Bu G. Apolipoprotein E and its receptors in Alzheimer’s disease: pathways, pathogenesis, and therapy. *Nat Rev Neurosci.* (2009) 10:333–44. 10.1038/nrn2620 19339974 PMC2908393

[58] Liu CC Liu CC Kanekiyo T Xu H Bu G. Apolipoprotein E and Alzheimer disease: risk, mechanisms, and therapy. *Nat Rev Neurol.* (2013) 9:106–18. 10.1038/nrneurol.2012.263 23296339 PMC3726719

[59] Phillips MC. Apolipoprotein E isoforms and lipoprotein metabolism. *IUBMB Life.* (2014) 66:616–23. 10.1002/iub.1314 25328986

[60] Prakashchand DD Mondal J. Conformational reorganization of apolipoprotein E triggered by phospholipid assembly. *J Phys Chem.* (2021) 125:5285–95. 10.1021/acs.jpcb.1c03011 33979165

[61] Johnson LA Olsen RH Merkens LS DeBarber A Steiner RD Sullivan PM Apolipoprotein E-low density lipoprotein receptor interaction affects spatial memory retention and brain ApoE levels in an isoform-dependent manner. *Neurobiol Dis.* (2014) 64:150–62. 10.1016/j.nbd.2013.12.016 24412220 PMC3936477

[62] Dietschy JM Turley SD. Thematic review series: brain lipids. Cholesterol metabolism in the central nervous system during early development and in the mature animal. *J Lipid Res.* (2004) 45:1375–97. 10.1194/jlr.R400004-JLR200 15254070

[63] Chang TY Yamauchi Y Hasan MT Chang C. Cellular cholesterol homeostasis and Alzheimer’s disease. *J Lipid Res.* (2017) 58:2239–54. 10.1194/jlr.R075630 28298292 PMC5711498

[64] Bian F Yang X Zhou F Wu PH Xing S Xu G C-reactive protein promotes atherosclerosis by increasing LDL transcytosis across endothelial cells. *Br J Pharmacol.* (2014) 171:2671–84. 10.1111/bph.12616 24517733 PMC4009008

[65] Li HY Liu XL Liu YT Jia ZK Filep JG Potempa LA Matrix sieving-enforced retrograde transcytosis regulates tissue accumulation of C-reactive protein. *Cardiovasc Res.* (2019) 115:440–52. 10.1093/cvr/cvy181 29992240

[66] Mookerjea S Francis J Hunt D Yang CY Nagpurkar A. Rat C-reactive protein causes a charge modification of LDL and stimulates its degradation by macrophages. *Arterioscler Thromb.* (1994) 14:282–7. 10.1161/01.atv.14.2.282 8305421

[67] Shih HH Zhang S Cao W Hahn A Wang J Paulsen JE CRP is a novel ligand for the oxidized LDL receptor LOX-1. *Am J Physiol Heart Circ Physiol.* (2009) 296:H1643–50. 10.1152/ajpheart.00938.2008 19252093

[68] Eisenhardt SU Starke J Thiele JR Murphy A Björn Stark G Bassler N Pentameric CRP attenuates inflammatory effects of mmLDL by inhibiting mmLDL–monocyte interactions. *Atherosclerosis.* (2012) 224:384–93. 10.1016/j.atherosclerosis.2012.07.039 22901456

[69] Paul A Ko KW Li L Yechoor V McCrory MA Szalai AJ C-reactive protein accelerates the progression of atherosclerosis in apolipoprotein E-deficient mice. *Circulation.* (2004) 109:647–55. 10.1161/01.CIR.0000114526.50618.24 14744975

[70] Kovacs A Tornvall P Nilsson R Tegnér J Hamsten A Björkegren J. Human C-reactive protein slows atherosclerosis development in a mouse model with human-like hypercholesterolemia. *Proc Natl Acad Sci USA.* (2007) 104:13768–73. 10.1073/pnas.0706027104 17702862 PMC1959457

[71] Teupser D Weber O Rao NT Sass K Thiery J Fehling HJ. No reduction of atherosclerosis in C-reactive protein (CRP)-deficient mice. *J Biol Chem.* (2011) 286:6272–9. 10.1074/jbc.M110.161414 21149301 PMC3057833

[72] Hirschfield GM Gallimore JR Kahan MC Hutchinson WL Sabin CA Benson GM Transgenic human C-reactive protein is not proatherogenic in apolipoprotein E-deficient mice. *Proc Natl Acad Sci USA.* (2005) 102:8309–14. 10.1073/pnas.0503202102 15919817 PMC1149444

[73] Schwedler SB Amann K Wernicke K Krebs A Nauck M Wanner C Native C-reactive protein (CRP) increases, whereas modified CRP reduces atherosclerosis in ApoE-knockout-mice. *Circulation.* (2005) 112:1016–23. 10.1161/CIRCULATIONAHA.105.556530 16087790

[74] Schwedler SB Kuhlencordt PJ Ponnuswamy PP Hatiboglu G Quaschning T Widder J Native C-reactive protein induces endothelial cell dysfunction in ApoE-/- mice: implications for iNOS and reactive oxygen species. *Atherosclerosis.* (2007) 195:e76–84. 10.1016/j.atherosclerosis.2007.06.013 17669410

[75] Schwedler SB Hansen-Hagge T Reichert M Schmiedeke D Schneider R Galle J Monomeric C-reactive protein decreases acetylated LDL uptake in human endothelial cells. *Clin Chem.* (2009) 55:1728–31. 10.1373/clinchem.2009.125732 19617288

[76] Narkates AJ Volanakis JE. C-reactive protein binding specificities: artificial and natural phospholipid bilayers. *Ann N Y Acad Sci.* (1982) 389:172–82. 10.1111/j.1749-6632 7046574

[77] Tsujimoto M Inoue K Nojima S. Reactivity of human C-reactive protein with positively charged liposomes. *J Biochem.* (1981) 90:1507–14. 10.1093/oxfordjournals.jbchem.a133617 7338519

[78] Bhakdi S Torzewski M Klouche M Hemmes M. Complement and atherogenesis: binding of CRP to degraded, nonoxidized LDL enhances complement activation. *Arterioscler Thromb Vasc Biol.* (1999) 19:2348–54. 10.1161/01.atv.19.10.2348 10521363

[79] Chang MK Binder CJ Torzewski M Witztum JL. C-reactive protein binds to both oxidized LDL and apoptotic cells through recognition of a common ligand: phosphorylcholine of oxidized phospholipids. *Proc Natl Acad Sci USA.* (2002) 99:13043–8. 10.1073/pnas.192399699 12244213 PMC130583

[80] Bhakdi S Torzewski M Paprotka K Schmitt S Barsoom H Suriyaphol P Possible protective role for C-reactive protein in atherogenesis: complement activation by modified lipoproteins halts before detrimental terminal sequence. *Circulation.* (2004) 109:1870–6. 10.1161/01.CIR.0000124228.08972.26 15037531

[81] Wang MS Messersmith RE Reed SM. Membrane curvature recognition by C-reactive protein using lipoprotein mimics. *Soft Matter.* (2012) 8:7909–18. 10.1039/C2SM25779C 24027600 PMC3767169

[82] Carson SD Ross SE. Effects of lipid-binding proteins apo A-I, apo A-IL, beta 2-glycoprotein I, and C-reactive protein on activation of factor X by tissue factor–factor VIIa. *Thromb Res.* (1988) 50:669–78. 10.1016/0049-3848(88)90325-8 3137684

[83] Dennis MW Downey C Brufatto N Nesheim ME Stevenson K Toh CH. Prothrombinase enhancement through quantitative and qualitative changes affecting very low-density lipoprotein in complex with C-reactive protein. *Thromb Haemost.* (2004) 91:522–30. 10.1160/TH03-08-0548 14983228

[84] Nesheim M Samis J Walker J Becker L Brufatto N Fischer T Lipoprotein-complexed C-reactive protein and the biphasic transmittance waveform in critically ill patients. *Blood Rev.* (2002) 16 Suppl 1:S15–22. 12918783

[85] Hulman G. The pathogenesis of fat embolism. *J Pathol.* (1995) 176:3–9. 10.1002/path.1711760103 7616354

[86] Rowe IR Soutar AK Pepys MB. Agglutination of intravenous lipid emulsion (’Intralipid’) and plasma lipoproteins by C-reactive protein. *Clin Exp Immunol.* (1986) 66:241–7. 3100118 PMC1542672

[87] Cheng Z Abrams ST Toh J Wang SS Downey C Ge X Complexes between C-reactive protein and very low-density lipoprotein delay bacterial clearance in sepsis. *J Immunol.* (2020) 204:2712–21. 10.4049/jimmunol.1900962 32269097

[88] Potempa LA Rajab IM Hart PC Bordon J Fernandez-Botran R. Insights into the use of C-reactive protein (CRP) as a diagnostic index of disease severity in COVID-19 infections. *Am J Trop Med Hyg.* (2020) 103:561–3. 10.4269/ajtmh.20-0473 32588812 PMC7410479

[89] Skotland T Sagini K Sandvig K Llorente A. An emerging focus on lipids in extracellular vesicles. *Adv Drug Deliv Rev.* (2020) 159:308–21. 10.1016/j.addr.2020.03.002 32151658

[90] Crawford JR Trial J Nambi V Hoogeveen RC Taffet GE Entman ML. Plasma levels of endothelial microparticles bearing monomeric c-reactive protein are increased in peripheral artery disease. *J Cardiovasc Transl Res.* (2016) 9:184–93. 10.1007/s12265-016-9678-0 26891844 PMC4874871

[91] Habersberger J Strang F Scheichl A Htun N Bassler N Merivirta RM Circulating microparticles generate and transport monomeric C-reactive protein in patients with myocardial infarction. *Cardiovasc Res.* (2012) 96:64–72. 10.1093/cvr/cvs237 22798388

[92] Pepys MB Hirschfield GM Tennent GA Gallimore JR Kahan MC Bellotti V Targeting C-reactive protein for the treatment of cardiovascular disease. *Nature.* (2006) 440:1217–21. 10.1038/nature04672 16642000

[93] Singh SK Agrawal A. Functionality of C-reactive protein for atheroprotection. *Front Immunol.* (2019) 10:1655. 10.3389/fimmu.2019.01655 31379851 PMC6646712

[94] Pathak A Singh SK Thewke DP Agrawal A. Conformationally altered C-reactive protein capable of binding to atherogenic lipoproteins reduces atherosclerosis. *Front Immunol.* (2020) 11:1780. 10.3389/fimmu.2020.01780 32849641 PMC7431523

[95] Cheng B Lv J-M Liang Y-L Zhu L Huang X-P Li H-Y Secretory quality control constrains functional selection-associated protein structure innovation. *Comm Biol.* (2022) 5:268–78. 10.1038/s42003-022-03220-3 35338247 PMC8956723

[96] Badimon L Peña E Arderiu G Padró T Slevin M Vilahur G C-reactive protein in atherothrombosis and angiogenesis. *Front Immunol.* (2018) 9:430. 10.3389/fimmu.2018.00430 29552019 PMC5840191

[97] Slevin M Krupinski J. A role for monomeric C-reactive protein in regulation of angiogenesis, endothelial cell inflammation and thrombus formation in cardiovascular/cerebrovascular disease? *Histol Histopathol.* (2009) 24:1473–8. 10.14670/HH-24.1473 19760596

[98] Strang F Scheichl A Chen YC Wang X Htun NM Bassler N Amyloid plaques dissociate pentameric to monomeric C-reactive protein: a novel pathomechanism driving cortical inflammation in Alzheimer’s disease? *Brain Pathol.* (2012) 22:337–46. 10.1111/j.1750-3639.2011.00539.x 21951392 PMC8092962

[99] Slevin M Matou-Nasri S Turu MM Luque A Rovira N Badimon L Modified C-reactive protein is expressed by stroke neo-vessels and is a potent activator of angiogenesis in vitro. *Brain Pathol.* (2010) 20:151–65. 10.1111/j.1750-3639.2008.00256.x 19170684 PMC8094831

[100] Zhang Z Na H Gan Q Tao Q Alekseyev Y Hu J Monomeric C-reactive protein via endothelial CD31 for neurovascular inflammation in an ApoE genotype-dependent pattern: a risk factor for Alzheimer’s disease? *Aging Cell.* (2021) 20:e13501–23. 10.1111/acel.13501 34687487 PMC8590103

[101] Slevin M Heidari N Azamfirei L. Monomeric C-reactive protein: current perspectives for utilization and inclusion as a prognostic indicator and therapeutic target. *Front Immunol.* (2022) 13:866379. 10.3389/fimmu.2022.866379 35309334 PMC8930844

[102] Heneka MT Carson MJ El Khoury J Landreth GE Brosseron F Feinstein DL Neuroinflammation in Alzheimer’s disease. *Lancet Neurol.* (2015) 14:388–405. 10.1016/S1474-4422(15)70016-5 25792098 PMC5909703

[103] Feringa FM van der Kant R. Cholesterol and Alzheimer’s disease; from risk genes to pathological effects. *Front Aging Neurosci.* (2021) 13:690372. 10.3389/fnagi.2021.690372 34248607 PMC8264368

[104] Tao Q Alvin Ang TF Akhter-Khan SC Itchapurapu IS Killiany R Zhang X Alzheimer’s disease neuroimaging initiative. impact of C-reactive protein on cognition and alzheimer disease biomarkers in homozygous apolipoprotein e ε4 carriers. *Neurology.* (2021) 97:e1243–52. 10.1212/WNL.0000000000012512 34266923 PMC8480484

[105] Gewurz H Mold C Siegel J Fiedel B. C-reactive protein and the acute phase response. *Adv Intern Med.* (1982) 27:345–72. 7041546

[106] Pathak A Agrawal A. Evolution of C-reactive protein. *Front Immunol.* (2019) 10:943. 10.3389/fimmu.2019.00943 31114584 PMC6503050

[107] Potempa LA Yao Z-Y Ji S-R Filep JG Wu Y. Solubilization and purification of recombinant modified C-reactive protein from inclusion bodies using reversible anhydride modification. *Biophys Rep.* (2015) 1:18–33. 10.1007/s41048-015-0003-2 26942216 PMC4762138

[108] Lv JM Chen JY Liu ZP Yao ZY Wu YX Tong CS mCellular folding determinants and conformational plasticity of native c-reactive protein. *Front Immunol.* (2020) 11:583–90. 10.3389/fimmu.2020.00583 32296446 PMC7137756

[109] Narkates AJ Volanakis JE. C-Reactive protein binding specificities: artificial and natural phospholipid bilayers. *Ann N Y Acad Sci.* (1982) 389:172–82. 10.1111/j.1749-6632 7046574

[110] Volanakis JE Narkates AJ. Binding of human C4 to C-reactive protein-pneumococcal C-polysaccharide complexes during activation of the classical complement pathway. *Mol Immunol.* (1983) 20:1201–7. 10.1016/0161-5890(83)90143-8 6558418

[111] Richards RL Gewurz H Osmand AP Alving CR. Interactions of C-reactive protein and complement with liposomes. *Proc Natl Acad Sci USA.* (1977) 74:5672–6. 10.1073/pnas.74.12.5672 271994 PMC431855

[112] McFadyen JD Zeller J Potempa LA Pietersz GA Eisenhardt SU Peter K. C-reactive protein and its structural isoforms: an evolutionary conserved marker and central player in inflammatory diseases and beyond. *Subcell Biochem.* (2020) 94:499–520. 10.1007/978-3-030-41769-7_20 32189313

