# Supplementary material for: Relevance of lipoproteins, membranes, and extracellular vesicles in understanding C-reactive protein biochemical structure and biological activities
Source: Front Cardiovasc Med. 2022 Sep 8;9:979461. doi: 10.3389/fcvm.2022.979461 (PMC9493015; doi:10.3389/fcvm.2022.979461)
Supplement: Supplementary file 1 [file Data_Sheet_1.docx]

**Supplementary information**

**Relevance of lipoproteins, membranes, and extracellular vesicles in understanding C-reactive protein (CRP) biochemical structure and biological activities**

**Lawrence A. Potempa**^1^**, Wei Qiao Qiu**^2, 3, 4^**, Ashley Stefanski**^1^**, Ibraheem M. Rajab**^1^

***Compendium of CRP purification methods and procedures from 1941 to present:***

The age of C-reactive protein research began in 1941 when Abernathy and Avery determined the substance from acute phase serum that precipitated with a pneumococcal cell wall extract described by Tillet & Francis (1930) was a heat-labile protein. Initial isolation of the “C-substance reactive protein” (i.e., CRP) used ammonium sulfate precipitation methods and reported lipids co-isolated with CRP. If lipids were removed, CRP behaved as a water-soluble albumin rather than a salt-soluble globulin protein (MacLeod & Avery 1941).

More than a decade later, Wood et al. (1954) refined the CRP isolation process using chloroform to partially delipidate serum before using calcium dependent precipitation with a C-polysaccharide fraction of the pneumococcal bacteria cell wall (i.e., CPS). The isolated CRP had beta electrophoretic mobility using free-zone electrophoresis, but gamma mobility when using a starch matrix. Even after treatment with chloroform, β-lipoproteins were found to co-isolate with CRP. The different electrophoretic mobilities noted for various CRP preparations are, in retrospect, a clue as to the nature of CRP as a pure protein or as a protein bound to lipoprotein particles.

Hokama & Riley (1963) next added DEAE anion exchange chromatography to ammonium sulfate fractionated serum to purify CRP. Protein that bound to this cationic solid phase was eluted using both calcium-chelating citrate buffers and a sodium chloride salt gradient. Recovered CRP bound comparatively stronger to DEAE than contaminating factors and displayed a predominant gamma electrophoretic mobility. While the strong bonding to the positively charged DEAE resin was consistent with its reported isoelectric point of 4.82 (Wood et al. 1954), its gamma electrophoretic mobility was in contradiction to this pI (gamma migrating proteins have neutral or slightly basic pIs), and its reported albumin-like solubility (being soluble in water rather than salt solutions). Weakly positive undefined protein migrating in both pre-albumin and albumin electrophoretic zones were noted in purified CRP fractions.

A more detailed understanding of CRP structure began in 1965 when Gotschlich & Edelman used mild alkali and chaotrope treatment to denature chloroform-extracted CRP preparations. Both major and minor bands of CRP were observed on starch gel electrophoresis. When analyzed by ultracentrifugation, isolated CRP was a heterogenous protein with a molecular weight from 110 kD to 140 kD. When denatured with 8 M urea or 5 M GuHCl, only a single, symmetrical protein with a size approximating an immunoglobulin light chain (~ 20 kD) was identified. The putative relationship to Ig light chains was further verified using starch gel electrophoresis in 8 M urea. When ultracentrifugation was repeated in 5 M GuHCl, using a partial specific volume of 0.735 as calculated from the amino acid content of CRP, a more precise molecular weight for the homogeneous subunit was calculated as 24.3 kD.

When non-denatured CRP was subjected to molecular sieve chromatography in mild alkaline conditions at pH 9.5, an asymmetrical bimodal elution pattern was observed, loosely describing three recovered fractions: 1.) CRP aggregates (i.e., having a Mw >160 kD), 2.) CRP multimers (i.e., having a Mw ~ 118 kD), and 3.) CRP subunits (i.e., having a Mw ~ 21.5 kD). The CRP aggregate was described as a highly denatured form of CRP, while the CRP multimer, which represented the majority of recovered protein sample (52%), displayed strong precipitation reactivity with CPS and strong reactivity with polyclonal anti-CRP antiserum. The CRP subunit fraction (representing 40% of the recovered protein sample), did not react with CPS but did show weak reactivity with the polyclonal anti-CRP reagent used. These results indicated that in mild basic conditions, a partial dissociation of CRP occurs, but protein remains soluble and multimeric. Gotschlich & Edelman reported no proteolytic peptides were noted when CRP was denatured, and that no carbohydrate or phosphate was found associated with either multimeric or subunit fractions of CRP.

When unfractionated CRP or the CRP 118kDa multimer fraction were subjected to starch gel electrophoresis at the alkaline pH of 9.3, 6 bands of various electrophoretic mobility were observed. Differently, when the CRP subunit fraction was subjected to this electrophoresis, only the two fastest anodally migrating bands were observed. If any of these three samples were first denatured in 8 M urea prior to electrophoresis, all samples produced identical banding patterns showing a single band with common mobility. In these early days of structural analysis, CRP was found to be a protein of heterogeneous sizes and electrophoretic mobilities, but that these different forms could be normalized into a common subunit sized protein.

Using various nucleotides and carbohydrate polymers such as DNA, RNA, dextran sulfate, heparin and cellulose, Gotschlich & Edelman (1967) reported CRP was a protein with a calcium-dependent affinity for phosphate monoesters. As an expanded understanding of its binding ligands evolved, advances and refinements in isolation and structural analyses ensued.

Kushner & Somerville (1970) used ammonium sulfate precipitation and citrate dialysis to prepare a CRP rich fraction from serum. When analyzed by gel filtration and density gradient centrifugation, they reported CRP had a sedimentation value of (Sw^20^) 6.6, a Stokes radius of 48.4 Å and an approximate molecular weight in serum of 135 kD-140 kD. They concluded CRP was composed of six (6) subunits, each with a Mw of 23,000. These authors noted asymmetrical elution and sedimentation patterns of CRP, suggesting that two forms of this protein, including a possible pentamer, might exist in serum. Taken together, the first 30 years of CRP research described it as a protein found in multiple sizes in serum, with various electrophoretic mobilities. When subjected to rigorous purification methods, all fractionated sizes of CRP could be reduced to a common subunit sized proteins of Mw ~ 23 kD suggesting the different forms of CRP in serum were either multimers of this subunit or were complexed with other molecules. An overview of various CRP purification methods used, and key observations made are summarized in **Table S1**.

Purification focused on lipid interactions.

Using knowledge that lipids and calcium had influence on CRP found in serum, Hokama et al. (1974) added lecithin to whole serum or to liver extracts. They observed a flocculent precipitate formed as a function of calcium, and that the precipitate contained CRP. After washing in water containing calcium, samples were resuspended in citrate saline solution and delipidated with chloroform prior to DEAE anion exchange and molecular sieve chromatography. CRP isolated in this way produced fractions with both gamma and beta electrophoretic mobility. CRP isolated from liver extracts additionally showed a protein with alpha electrophoretic mobility. Using immunochemical precipitation techniques, these differently migrating forms of CRP showed partial immunological identity using polyclonal anti-CRP reagents. Of retrospective relevance to immunological cross-reactivity using polyclonal reagents, anti-CRP antiserums have been shown to contain significant specificity to both the antigenically distinct pCRP and mCRP isoforms (Potempa et al. 2015).

Kaplin & Volanakis (1974) used each of pneumococcal cell wall C polysaccharide fraction (CPS), choline phosphatides, lecithin, and sphingomyelin to precipitate CRP from serum. Isolated protein had weak antigenic reactivity to albumin and α1-globulins and showed a relationship to the C1q component of the classical complement pathway. No immunoglobulin proteins were observed in the CRP-containing precipitates. The CRP-CPS precipitation reaction could be inhibited by haptenic phosphocholine suggesting PC groups in the Gram-positive teichoic acid fraction of CPS was a primary ligand in this interaction.

Purification methods that focused on lipid-free CRP.

Osmand et al. (1975) covalently linked CPS to an agarose-based resin for affinity isolation of CRP from pleural or ascites fluids. As a first step, column feeds were delipidated and clarified using celite. By including 2 mM calcium in the feed and washing steps, CRP bound the phosphocholine groups exposed on immobilized CPS. CRP was recovered in high yield as a soluble, concentrated protein with a high degree of purity when calcium was chelated using citrate buffers at pH 8.0. As a final purification step to separate CRP aggregates and subunits and a small amount of contaminating IgG, affinity purified CRP was further processed by molecular sieve chromatography. The delipidated, affinity purified CRP had an apparent Mw mid-way between IgG (~160kDa) and albumin (Mw 68 kD), a predominant subunit protein band of 23.5 kD (as analyzed by SDS-PAGE), and a gamma electrophoretic mobility. Minor protein components of slightly smaller molecular weight were often seen using denaturing electrophoresis.

As purification and analytical methods were refined, CRP entered an era of detailed structure/function analyses. Using an ammonium sulfate/calcified water/chloroform/CPS precipitation method, Oliveira et al. (1977) reported CRP to be a homopolymer composed of subunits having 187 amino acids and ~21 kD size. Using their advanced understanding of CRP’s affinity for exposed PC groups, they went on to synthesize their own affinity resin covalently binding PC ligands to an agarose resin using extended linear spacers of amide linked hexyl and caproyl-PC groups, thus avoiding the need to isolate the C-polysaccharide substance from Gram-positive bacteria. While calcium was required for CRP binding to the affinity resin, this group used a gradient of haptenic PC to recover bound protein. This approach minimized any protein structural changes that may have been elicited by removal of calcium ions from the PC binding sites. Of note, these authors also reported an alternative binding specificity of CRP for N-acetyl galactosamine residues.

Purification methods focused on calcium supplementation and chelation.

Young & Williams (1978) and Pontet et al. (1978) also used PC affinity columns to isolate CRP from ascites fluids. The level of calcium supplementation to starting and washing fluids (varied from 1 mM to 10 mM) and the elution method used (using calcium-containing haptenic PC ligand or calcium chelating reagents) were not originally considered to be significant variables in the isolation protocols. As calcium binding residues in CRP were defined, and as calcium binding was shown to affect and regulate the PC binding site on each subunit, little attention was paid to how calcium affected packing of the pentameric protein. It is now known various levels of calcium do influence pentameric packing, with CRP compacting from an apparent size of 115kD in 2 mM calcium (i.e., physiological calcium levels) to a protein of apparent size of 86 kD in 10 mM calcium (Rajab et al. 2020a). Conversely, in strongly chelating citrate or EDTA buffers, the apparent size of CRP increases to an apparent size of 133 kD.

Calcium has been reported to be a critical factor in stabilizing the CRP exposed to a variety of denaturing conditions, and in preventing it from being proteolyzed (Potempa et al. 1983; Kinoshita et al. 1989). Calcium ions thus act not only as a regulator of CRP PC binding activity, but as a constrictor of protein packing as a type of tertiary and quaternary structural glue. An awareness of how different calcium levels affect the structural packing of CRP and the accessibility of (all) its binding sites becomes important variables in understanding how CRP interacts with ligands. To this end, that CRP purified by Young & Williams (using very high calcium concentrations) did not cause β-lipoprotein coated erythrocytes to agglutinate, adding question to the significance of reported CRP β-lipoprotein interactions. A reassessment of how calcium supplementation affected these results is warranted.

Elaboration of the structural coordinates of CRP as a lipid free protein.

As molecular biology techniques using genomic analyses were advanced, Lei et al. (1985) and Woo et al. (1985) reported the CRP subunit primary sequence contained 19 amino acids that were not included the sequence first published by Oliveira. This added sequence inserted after residue 64 (i.e., extending from residues 65-84 of the currently established 206 amino acid primary sequence) and involves residues contributing to calcium ion and PC binding sites. Three dimensional structural analyses of the purified protein (Shrive et al. 1996) further established CRP as a non-glycosylated, homogeneous multimer of five globular subunits, arranged non-covalently in a cyclic pentameric discoid orientation with a central void. The established quaternary structure shows that PC and calcium binding sites of the pentameric disc all localized to one face of the doughnut-shaped structure, suggesting that when the pentamer binds to surfaces presenting accessible PC ligands, the annular disk sits flat on the surface with the opposite pentameric face exposed so that effector systems involved in CRP bioactivity (e.g., complement, leukocytes) can be engaged. While contributing important detail to locations and biochemical interactions of all residues that describe CRP as a rigid pentameric protein, it is important that experimental interpretations of CRP do not solely focus on these 3-D snapshots as absolute. By minimizing or ignoring structural fluctuations that could occur when CRP binds its ligands or is otherwise modified by local environmental conditions, subunit dissociation, or group modifications, data interpretation can be prejudiced. Furthermore, and as relevant to this review, early descriptions of CRP in blood often included reference to lipid associations. Selective visualization of CRP as an isolated rigid protein limit thinking on how lipid interactions might affect various levels of protein structure and contribute to distinctive CRP bioactivities.

**Table S1: Purification Methods Used to Isolate CRP from Body fluids**

| Experimental focus of publication | Isolation methods used | Purification notes | References |
| --- | --- | --- | --- |
| Pneumococcal cell wall reactive substance in acute phase serum was a heat labile protein | Ammonium sulfate | - Lipids co-isolated with the protein - When lipids were removed, the protein behaved as an albumin and not a globulin | Abernethy & Avery 1941  MacLeod & Avery 1941 |
| Precipitation with pneumococcal C-polysaccharide (CPS) | Delipidated with chloroform  Calcium-dependent precipitation with CPS | - Even with chloroform extraction, β-lipoproteins co-isolated - Protein migrated using SPE in both beta and gamma zones - Isoelectric point = 4.82 | Wood et al. 1954 |
|  | Ammonium sulfate  DEAE anion exchange chromatography | - Bound strongly to DEAE resin - Eluted with salt gradient - Migrated as a gamma globulin (in apparent contradiction to Wood et al.) - Some CRP was observed in albumin and pre-albumin SLE zones | Hokama & Riley 1963 |
| Detailed CRP structure | Used chloroform extracted serum | - Used denaturation techniques to identify CRP subunits of 24.3 kD - Identified major and minor bands on SPE - Used non-denaturing molecular sieve chromatography to identify 3 fractions aggregates (> 160 kD), multimers (~ 118 kD) and subunits (~ 21.5 kD). Subunit fraction did not react with CPS. - Multimer fraction could be changed into subunit fraction with urea denaturation | Gotschlich & Edelman 1965 |
| CRP binding ligands |  | - Identified CRP as having calcium -dependent affinity for phosphate monoesters | Gotschlich & Edelman 1967 |
| CRP purification | Barium sulfate precipitation | - Protein had both pre and post albumin mobilities on SPE | Ganrot & Kindmark 1969 |
| CRP purification and characterization | Ammonium sulfate  Citrate dialysis  Gel filtration  Density gradient centrifugation | - Identified CRP as a hexamer - Observed asymmetrical elution   and sedimentation patterns suggesting some protein may be pentameric | Kushner & Somerville 1970 |
| CRP purification focused on lipids | Added lecithin to whole serum or liver extracts  Then delipidated with chloroform and passed down anion exchange and molecular sieve chromatography | - Identified CRP in a precipitate - Reported two forms by SPE (a gamma and a beta globulin) - Liver extracts also produced an alpha-globulin migrating form - All forms reacted with polyclonal anti-CRP reagents | Hokama et al. 1974 |
| Added CRP binding ligands to serum as to influence purification | Used CPS, choline phosphatides, lecithin, sphingomyelin to precipitate CRP | - Excluded Igs as-co-precipitating factors - Identified a relationship of CRP for C1q of complement | Kaplan & Volanakis 1974 |
| Synthesized a CRP affinity resin to purify CRP | Used celite to remove lipids from starting fluids  Used calcium to bind and citrate to elute  Collected protein further purified by molecular sieve chromatography | - Did identify aggregates, subunits, and some IgG in purified CRP fractions - Purified protein migrated as a gamma globulin in SPE | Osmond et al 1975 |
| Factors of interest in Hodgkin’s Disease | Used spleen extract and DEAE anion exchange chromatography | - Found CRP and C1q co-isolated with beta lipoproteins - Suggested apolipoprotein B as a ligand of interest in studies of CRP | Bieber et al. 1977  Olofsson et al. 1987 |
| Further use of affinity resins | Synthesized phosphocholine-derivatized agarose resin | - Eluted using hapten PC (avoiding use of calcium-chelating buffers and reagents) - Thus, purified CRP did not display binding to beta-lipoproteins | Young & Williams 1978 |
| Affinity purification | Used 2-aminoethanol phosphate substituted agarose | - Used high calcium supplementation (10 mM) to bind, then washed calcium away before eluting with citrate gradient - Identified Serum Amyloid P component (SAP – a pentraxin analog of CRP) as a co-isolated factor | Pontet et al. 1978 |
| Affinity purification, anion exchange and molecular sieve chromatography | Added a Glycyl-tyrosine spacer to immobilize PC ligands to agarose resins | - Isolated CRP from ascites fluids using calcium-borate buffer, eluting CRP with EDTA. Clarified/delipidated ascites fluids using celite prior to column passage. - Collected CRP was further purified using anion exchange chromatography with salt gradient elution, and molecular sieve chromatography - Purified CRP = 6.5 S - SDS PAGE showed apparent Mw shifted to apparent high Mw when protein was reduced and alkylated | Volanakis et al. 1978 |
| Affinity purification, anion exchange and molecular sieve chromatography |  | - Binding to membrane PC was maximized when diacyl lipid was se-esterized into mono-acyl lecithin (lysolecithin) | Volanakis & Wirtz 1979 |
|  |  | - CRP binds phosphate monoesters with relatively low affinity Ka = 2-3 x 10^4^ M ^-1^ - Affinity increases to 1.6 x 10^5^ M^-1^ when positively charged choline is present - Other binding ligands include Nucleotides, DNA, RNA, and chromatin | Liu et al. 1982  Oliviera et al. 1980  Gotschlich & Edelman 1967  Robey et al. 1984 |
| Affinity purification | Extended the linker used in immobilizing PC groups to agarose resins – used amide linked hexyl and caproyl groups  Eluted CRP in the presence of calcium using hapten PC | - Identified histidine residues are involved in the calcium-regulated binding of CRP to PC - Proposed CRP might also bind N-acetyl galactosamine |  |
| CRP hepatic synthesis |  | - CRP is synthesized in the liver and is stored in vesicles prior to release. VLDL and apo B co-localize with CRP in secretory vesicles | Kushner & Feldman 1978 |

**Supplementary material references:**

Abernethy TJ, Avery OT. 1941. The occurrence during acute infections of a protein not normally present in the blood: I. Distribution of the reactive protein in patients' sera and the effect of calcium on the flocculation reaction with C polysaccharide of pneumococcus. J Exp Med 73(2):173-182. <http://doi:10.1084/jem.73.2.173>

Bieber MM, Fuks Z, Kaplan HS. 1977. E-rosette inhibiting substance in Hodgkin's disease spleen extracts. Clin Exp Immunol 29(3):369-375.

Ganrot PO, Kindmark CO. 1969. A simple two-step procedure for isolation of C-reactive protein. Biochim Biophys Acta 194(2):443-448. <http://doi:10.1016/0005-2795(69)90104-4>

Gotschlich EC, Edelman GM. 1965. C-reactive protein: a molecule composed of subunits. Proc Natl Acad Sci USA 54(2):558-566. <http://doi:10.1073/pnas.54.2.55>

Gotschlich EC, Edelman GM. 1967. Binding properties and specificity of C-reactive protein. Proc Natl Acad Sci USA 57(3):706-712. <http://doi:10.1073/pnas.57.3.706>

Hokama Y, Riley RF. 1963. Purification of C-reactive protein, an acute phase protein of human serum. Biochim Biophys Acta 74:305-308. <http://doi:10.1016/00063002(63)91371-4>

Hokama Y, Tam R, Hirano W, Kimura L. 1974. Significance of C-reactive protein binding by lecithin: a simplified procedure for CRP isolation. Clin Chim Acta 50(1):53-62. <http://doi:10.1016/0009-8981(74)90077-1>

Kaplan MH, Volanakis JE. 1974. Interaction of C-reactive protein complexes with the complement system I. Consumption of human complement associated with the reaction of C-reactive protein with Pneumococcal C-polysaccharide and with choline phosphatides, lecithin, and sphingomyelin. J Immunol 112:2135-2147

Kinoshita CM, Ying S-C, Hugli TE, Siegel JN, Potempa LA, Jiang H, Houghton RA, Gewurz H. 1989. Elucidation of a protease-sensitive site involved in the binding of calcium to C-reactive protein. Biochemistry 28:9840-9848 <http://doi:10.1021/bi00451a044>

Kushner I, Somerville JA. 1970. Estimation of the molecular size of C-reactive protein and Cx-reactive protein in serum. Biochim Biophys Acta 207(1):105-114. <http://doi:10.1016/0005-2795(70)90140-6>

Lei KJ, Liu T, Zon G, Soravia E, Liu TY, Goldman ND. 1985. Genomic DNA sequence for human C-reactive protein. J Biol Chem 260(24):13377-13383.

[Liu](https://nyaspubs.onlinelibrary.wiley.com/action/doSearch?ContribAuthorStored=Liu%2C+Teh-Yung) T-Y, Robey FA, Wang C-M. 1982. Structural studies on C-reactive protein. Ann NY Acad Sci 389:151-162. <https://doi.org/10.1111/j.1749-6632.1982.tb22133.x>

Macleod CM, Avery OT. 1941. [The occurrence during acute infections of a protein not normally present in the blood : II. Isolation and properties of the reactive protein.](https://www.ncbi.nlm.nih.gov/pubmed/19871071) J Exp Med 73(2):183-190. <http://doi:10.1084/jem.73.2.183>

Oliveira EB, Gotschlich EC, Liu TY 1977. Primary structure of human C-reactive protein. Proc Nat Acad Sci 74(8):3148-3151. <http://doi:10.1073/pnas.74.8.3148>

Oliveira EB, Gotschlich C, Liu TY. 1979. Primary structure of human C-reactive protein. J Biol Chem 254(2):489-502.

Oliveira EB, Gotschlich EC, Liu TY. 1980. Comparative studies on the binding properties of human and rabbit C-reactive proteins. J Immunol 124(3):1396-1402.

Olofsson SO, Bjursell G, Boström K, Carlsson P, Elovson J, Protter AA, Reuben MA, Bondjers G. 1987. Apolipoprotein B: structure, biosynthesis, and role in the lipoprotein assembly process. Atherosclerosis 68(1-2):1-17. <http://doi:10.1016/0021-9150(87)90088-8>

Osmand AP, Mortensen RF, Siegel J, Gewurz H. 1975. Interactions of C-reactive protein with the complement system. III. Complement-dependent passive hemolysis initiated by CRP. J Exp Med 142(5):1065–1077. <https://doi.org/10.1084/jem.142.5.1065>

Pontet M, Engler R, Jayle MF. 1978. One step preparation of both human C-reactive protein and C1t. FEBS Lett 15;88(2):172-175. <http://doi:10.1016/0014-5793(78)80167-7>

Potempa LA, Maldonado BA, Laurent P, Zemel E, Gewurz H. 1983. Antigenic, electrophoretic, and binding alterations of human C-reactive protein modified selectively in the absence of calcium. Mol. Immunol. 20: 1165-1175. <http://doi:10.1016/0161-5890(83)90140->

Rajab IM, Hart PC, Potempa LA. 2020a. How C-reactive protein structural isoforms with distinctive bioactivities affect disease progression. Frontiers Immunology – Molecular Innate Immunity 112:126-2138. <http://doi10.3389/fimmu.2020.02126>

Shrive AK, Cheetham GM, Holden D, Myles DA, Turnell WG, Volanakis JE, Pepys MB, Bloomer AC, Greenhough TJ. 1996. Three-dimensional structure of human C-reactive protein. Nat Struct Biol 3(4):346-354. <http://doi:10.1038/nsb0496-346>

Tillett WS, Francis T. 1930. Serological reactions in pneumonia with a non-protein somatic fraction of pneumococcus. J Exp Med 52(4):561-571. <http://doi:10.1084/jem.52.4.561>

Volanakis JE, Clements WL, Schrohenloher RE 1978. C-reactive protein: purification by affinity chromatography and physicochemical characterization. J Immuno Meth 23(3-4):285-295. <https://doi.org/10.1016/0022-1759(78)90203-X>

Volanakis JE, Wirtz KW. 1979. Interaction of C-reactive protein with artificial phosphatidylcholine bilayers. Nature 281(5727):155-157. <http://doi:10.1038/281155a0>

Woo P, Korenberg JR, Whitehead AS. 1985. Characterization of genomic and complementary DNA sequence of human C-reactive protein, and comparison with the complementary DNA sequence of serum amyloid P component. J Biol Chem. 260(24):13384-13388.

Woo P, Korenberg JR, Whitehead AS. 1985. Characterization of genomic and complementary DNA sequence of human C-reactive protein, and comparison with the complementary DNA sequence of serum amyloid P component. J Biol Chem. 260(24):13384-13388.

Wood HF, McCarty M, Slater RJ. 1954. The occurrence during acute infections of a protein not normally present in the blood. V. Physical-chemical properties of the C-reactive protein crystallized by a modified technique. J Exp Med 100(1):71-79. <http://doi:10.1084/jem.100.1.71>

Young NM, Williams RE. 1978. Comparison of the secondary structures and binding sites of C-reactive protein and the phosphorylcholine-binding murine myeloma proteins. J Immunol 121(5):1893-1898.
